# Which Choice of Delivery Model(s) Works Best to Deliver Fortified Foods?

**DOI:** 10.3390/nu11071594

**Published:** 2019-07-14

**Authors:** Baqir Lalani, Aurélie Bechoff, Ben Bennett

**Affiliations:** Natural Resources Institute, University of Greenwich. Medway Campus, Central Avenue, Chatham Maritime, Kent ME4 4TB, UK

**Keywords:** fortification, biofortification, complementary food, business model, cost effectiveness, micronutrient deficiency

## Abstract

Micronutrient deficiencies (MNDs) occur as a result of insufficient intake of minerals and vitamins that are critical for body growth, physical/mental development, and activity. These deficiencies are particularly prevalent in lower-and middle-income countries (LMICs), falling disproportionately on the poorest and most vulnerable segments of the society. Dietary diversity is considered the most effective method in reducing this deficiency but is often a major constraint as most foods rich in micronutrients are also expensive and thereby inaccessible to poorer members of society. In recent years, affordable commodities such as staple foods (e.g., cereals, roots, and tubers) and condiments (e.g., salt and oil) have been targeted as “vehicles” for fortification and biofortification. Despite efforts by many countries to support such initiatives, there have been mixed experiences with delivery and coverage. An important but little understood driver of success and failure for food fortification has been the range of business models and approaches adopted to promote uptake. This review examines the different models used in the delivery of fortified food including complementary foods and biofortified crops. Using a keyword search and pearl growing techniques, the review located 11,897 texts of which 106 were considered relevant. Evidence was found of a range of business forms and models that attempt to optimise uptake, use, and impact of food fortification which are specific to the ‘food vehicle’ and environment. We characterise the current business models and business parameters that drive successful food fortification and we propose an initial structure for understanding different fortification business cases that will offer assistance to future designers and implementors of food fortification programmes.

## 1. Introduction

Micronutrient deficiencies (MNDs) occur as a result of insufficient intake of minerals and vitamins that are critical for body growth, physical and mental development, and activity [1]. The main MNDs are iron, iodine, vitamin A, zinc, and folate. For example, it is estimated that 2 billion people suffer from iron deficiency anaemia (IDA) worldwide [2]. These deficiencies can affect individuals residing in low- and middle-income countries (LMICs) but also in higher income countries including not only those that are under-nourished but also those that are over-nourished and living on a poor quality diet. The impacts of MNDs often fall disproportionately on women and children because of the critical nutritional requirements during infancy childhood, pregnancy, and lactation [3,4,5]. Inadequate dietary practices including poor quality diet is considered one of the driving factors alongside other factors such as poverty, infectious diseases, and inadequate care [6]. 

The World Health Organization (WHO) and the United Nations Food and Agriculture Organization (FAO) have identified four main strategies for addressing micronutrient malnutrition: (a) nutrition education leading to increased diversity and quality of diets; (b) food fortification and biofortification; (c) supplementation; and (d) disease control measures. Each of these strategies has a place in reducing micronutrient malnutrition. For maximum impact, the appropriate mix of these strategies should be in place simultaneously. It is argued that the most effective intervention to alleviate micronutrient deficiency is through a diversified diet which includes fruit, vegetables, fish, and meat [3,5]. In many LMICs, this is currently impractical for large swaths of the populace given availability, access, and compliance issues, particularly for the poorest and most vulnerable segments of society. 

Many factors contribute to the success or failure in reducing micronutrient deficiency in LMICs and this review covers the factors relating to public and private sector commitment to food fortification (and biofortification). Drawbacks included weak governance, infrastructure, and insufficient funding [7]. In addition, a major constraint is that most foods that are rich in micronutrients also tend to be expensive and sometimes unaffordable to the poor who are most at risk of malnutrition. Therefore, affordable commodities such as staple foods (cereals, roots, and tubers) and condiments (salt and oil) have been targeted as “vehicles” (Fortification requires suitable food vehicles i.e. those foods that are widely consumed and therefore have the ability to reach/ accessed by the most amount of people)for fortification and biofortification [3,8]. Direct interventions to address MNDs range from: (i) use of supplements (in tablet/sachet form or liquid droplets), conventional fortification (where minerals or vitamins are added to processed foods, such as packaged cereals and flours), and biofortification of staple foods usually via conventional breeding and mineral rich fertiliser or genetic engineering. 

In 2008, the Copenhagen Consensus—a panel of expert economists that assessed the most cost- effective strategies for addressing global challenges worldwide—ranked different strategies (ex ante) to alleviate MNDs according to their cost-effectiveness [2]. Considering the private and public sector commitments, micronutrient supplements in vitamin A and zinc were ranked first in terms of costs vs. benefits. Micronutrient fortification (i.e., iron and salt iodization) ranked third after trade liberalisation (the Doha Development Agenda) and thereby considered one of the “best buys” [9], whilst biofortification ranked highly in fifth position just after expanded immunisation coverage for children. This positioning highlights that fortification and biofortification are amongst the most cost-effective strategies to reduce micronutrient deficiencies and hence malnutrition. Fortification can be undertaken at very large scale because the vehicles for fortification are commonly consumed commodities such as flour, salt, sugar, and oil. Fortification can therefore have a large national coverage reaching mostly urban and peri-urban areas. On the other hand, the main niche for biofortification is in rural areas where it is harder for fortified foods to be accessed [10]. According to Meenakshi et al. [11], biofortification is likely to be more costly in Africa than in Asia, due in part, to poorer seed systems in Africa and the likelihood of higher costs incurred due to lower population density. Notwithstanding, the demand for fortification and biofortification is projected to increase in spite of dietary shifts in the coming years [12]. Fortification and biofortification programmes are further expected to play a leading role alongside supplementation programmes in addressing micronutrient deficiency with an approach linking agriculture and nutrition [13]. 

Despite efforts by many countries to support such initiatives, there have been mixed experiences with delivery and coverage. An important but little understood driver of success and failure for food fortification and biofortification has been the range of business models (The terms business models and delivery models are used interchangeably throughout the manuscript.) (from public to private-led) and approaches adopted to promote uptake [14,15,16]. The term “business model” has been widely debated [17] but in principle has been defined as: “A specific combination of resources which through transactions generate value for customers and the organization” ([17] p 382). Neo-classical economics posits that market forces allocate resources and set equilibrium. In this theory, the firm intermediates between producer and consumer. Numerous sub-theories exist to explain how the firms organise themselves, operate, and interact. Notwithstanding the internal workings of firms are often considered something of a “black-box” in economic theory [18]. Important issues include: competitive advantage and how to outperform other firms [19] how value chains are formed and upgraded [20], internalisation and the inclusion or exclusion of core competencies [21], and the potential advantages of bringing different business elements together in clusters [22]. New technologies have opened the possibility of alternative business models with greater social inclusion, lower transaction costs, and faster start-up [23]. Businesses with purely social aims (e.g., “social enterprises”) follow the same fundamental theoretical under-current, but with an emphasis on measured social outcomes and impact instead of profit. Crucially, at the level of the individual firm, these entities too must realise income higher than costs or fail.

Given this as backdrop, the objective of this review is to examine the efficacy of different business models used in the delivery of fortified food including complementary foods (Complementary foods were defined here in a broader sense of food complementing the diet rather than in the strict sense of infant foods) and biofortified crops worldwide and to draw lessons for LMICs. The review describes the key features of sustainable, effective business models for the production of fortified foods and biofortified crops in LMICs.

The key hypothesis is that there are a range of business forms and models that optimise uptake, use and impact of food fortification of different kinds, in different environments. A primary aim of this review has been to develop and characterise the business models and business parameters that drive successful food fortification and to propose a typology. 

An explanation of the methodology is found in Section 2. Section 3 presents an overview of the themes and historical overview that has shaped the current fortification/biofortification landscape. Section 4 examines the different delivery models used. Section 5 provides concluding comments to the review and proposes an initial structure for understanding the different business cases and their impact on food fortification. 

## 2. Materials and Methods 

**Figure 1:**
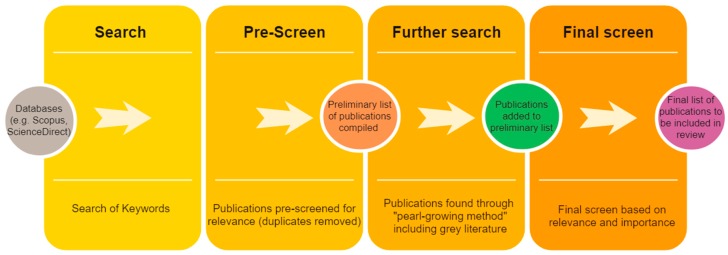
Diagram outlining review methodology used.

The review follows an adapted approach advocated by Cooper et al. [24]. Published studies (i.e., peer reviewed journal articles/book chapters) were identified through keyword searches in popular search engines i.e., ScienceDirect, Scopus, Google Scholar and Web of Science (Table 1) and subsequently through the “pearl growing” method [24] i.e., identifying highly cited literature from the existing literature sourced and key grey literature i.e., unpublished literature including online reports/project documents. Filtering was also administered according to the authors’ knowledge of the topic/purpose of the review and published literature was stored in Endnote version X9. In addition, the main catalogue/business literature of the British Library was also consulted as well as the main catalogue of the London School of Hygiene and Tropical Medicine (LSHTM) library. The full search procedure is described in Figure 1. 

Using the keyword search and the review located 11,897 texts of which 80 were considered relevant/accessible. An additional 53 articles/unpublished reports were gathered through pearl growing techniques. Following the final screen of the full texts (based on relevance and importance) we have included 106 in the final review. 98 peer reviewed published journal articles/book chapters and 8 unpublished documents including online reports. 

## 3. Fortification of Staple Crops, Complementary Foods, and Biofortification

We begin our review by considering the literature relating to the wider aspects of fortification of staple crops, complementary foods and biofortification. Fortification of food is approached in two main ways: by the addition of micronutrients to staple foods or other food vehicles such as complementary foods, and through biofortification which is the integration of micronutrients in crop varieties through breeding.

### 3.1. Fortification of Staple Crops and Complementary Foods 

Fortification is the practice of deliberately adding essential micronutrients to a food in order to provide a public health benefit by decreasing MNDs. Modern day fortification started in the early 20th century to target specific health conditions such as the aim to combat rickets with Vitamin D fortified milk [25]. These efforts have likely contributed to wide ranging benefits for well-being in industrialised countries [25]. The definition of fortification has also evolved in recent years. Definitions now include single as well as multiple nutrients added to food (e.g., triple fortification of salt) in addition to multiple nutrient supplementation also includes sprinkles—microencapsulated micronutrients that can be added to complementary food and other foods [25]. Despite many successes across the globe fortification of foodstuffs could have a much larger impact on reducing micronutrient deficiency, especially in LMICS, given low awareness/demand, less developed markets, and limited scope for centrally-processed food [25]. This is specifically the case for vehicles such as wheat flour and rice, which provide significant scope to increase the volume of fortification [26]. 

Fortification initiatives have largely been spearheaded by mandatory fortification efforts. This approach uses the force of the law to ensure the addition of certain micronutrients to certain products (typically staple foods such as flour, oil, salt, etc.) [27]. Whilst largely successful in many industrialised countries two major challenges remain: (i) the ability to guarantee that standards of nutrient quality are met, and, (ii) universal coverage through mass fortification not to exclude the most vulnerable members of society. Voluntary fortification efforts have also taken place, which are largely at the behest of businesses that choose voluntarily to add fortificants to food products [27]. This is because fortification often requires manufacturing and/or adequate distribution channels and the cost of adding these micronutrients need to be covered even if there is no additional cost to the consumer in the form of higher prices; this may thus exclude poor members of society from benefiting given the lack of investment in distribution [28]. Costs of fortification, therefore, need to be kept low in order for poorer members of society to benefit given the high sensitivity to price changes among this group and the effect this will have on consumption [29]. 

Where voluntarily fortification has taken place, this has often resulted in a failure to reach scale/target groups [30]. For example, in Ireland, though voluntary fortification had been used to increase the amount of folic acid (added to yogurt and breakfast cereals) in the Irish diet to target women of childbearing age who are more at risk, intake was lower among them [26]. Likewise, in the Philippines, voluntary fortification for staples (e.g., wheat flour and oil) led to products being out of reach for ordinary Filipino families because of the higher cost [26]. Some recent successes of voluntary fortification are evident though. Cargill, a US based family owned agricultural multi-national, has voluntarily fortified oil in India and has redesigned its brands to focus on the fortified oil’s health benefits. This has included successfully raising consumer awareness of its fortified product through marketing and word of mouth campaigns in addition to improving awareness among commercial distributors and sales agents of the health benefits of its fortified produce [31]. Likewise, successes with voluntary fortification have been found in South East Asia (e.g., Philippines and Cambodia) particularly with fortification of sauces, processed foods, and seasonings [12]. These have largely been successful, for example, where market share was high. In the Philippines, the addition of MSG (monosodium glutamate) a popular flavouring agent with controversial effects on health was deemed a successful case of voluntary fortification due to centralised production and there being only two manufacturers in the country with one controlling 90% of the market share [12]. There exists further scope to support fortification of processed food vehicles either directly or indirectly by using fortified staples in their production [12].

In contrast, a promising example of mandatory legislation has been seen with the case of salt iodisation (to tackle iodine deficiency that causes mental retardation and goiter) which has seen a rapid increase in household coverage (from 49% to 72%) in the decade following legislation in countries following mandatory legislation compared with more modest increase for those countries following voluntary iodisation (40% to 49%) [26]. Furthermore, concentration of milling is seen as an important factor. The successful case of the 77 countries that mandated wheat flour fortification, for example, showed that 90% of these had industrially milled flour compared to only 5% of flour (industrially milled) where countries had gone through fortification voluntarily [26]. Elhakim et al. [32] recently showed that the mandatory fortification of wheat flour (ferrous sulfate and folic acid) in Egypt has resulted in 50 million Egyptians now consuming quality assured fortified bread. Moreover, a systematic review by Pachón et al. [33] has also highlighted countries with mandatory fortified wheat and maize flour have had a significant impact in terms of reducing the prevalence of low ferritin. Greiner [34] also suggested that staple foods in particular should be fortified through mandatory legislation. The need for developing capacities in assessment and monitoring to ensure the right concentrations of nutrients are added are seen as a priority [34]. 

As of 2019, 132 countries have enacted mandatory legislation for mass fortification of either wheat, maize, or rice (see Tables S1–S3; Figures S1 and S2). Rwanda is the only exception as it has initiated voluntary fortification solely for maize flour (see Tables S1–S3). Examples of countries where high rates of industrially milled flour coupled with voluntary fortification do exist. These include China (89% industrially milled wheat flour), and low-income countries such as Afghanistan (92% industrially milled wheat flour) and Sierra Leone (100%) (see Tables S2 and S3). In addition, five countries (Democratic Republic of Congo, Gambia, Namibia, Qatar, and United Arab Emirates) have initiated voluntary fortification efforts with at least half of their wheat flour being industrially milled and fortified with iron and/or folic acid [35]. These countries, however, do have very limited wheat flour milling capacity and so an effective monopoly (De Groote, 2019, personal communication). This would arguably be more difficult in larger economies with bigger food processing sectors and more competition (De Grote, 2019 personal communication). 

Despite the success of such efforts, particularly mandatory legislation initiatives, it is estimated that for maize flour less than half (48%) is currently fortified [35]. This is of particular concern for maize consuming countries. Tanzania and Uganda are a case in point. For example, in Tanzania the proportion of the population found to be consuming fortified maize flour is still very low (only 2.5%). Likewise, only 6.5% of the populace in Uganda were found to be consuming fortified maize flour (See Table S3). One of the main challenges to reaching scale is the fact that households are often subsistence farms reliant on their own unfortified produce or consume locally produced maize meal or wheat flour which is not fortified and processed at the local level (often village-level) in smaller capacity mills e.g., small-scale hammer mills [35]. For example, in Uganda only a small proportion (30%) of the maize flour is industrially milled (See Table S3). The availability of mills with fortification technology significantly affects the ability to fortify maize and wheat flour in the particular country [35]. Fiedler et al. [36] show the high dependency on small scale grain mills (e.g., small scale hammer mills) in Uganda, Tanzania, and Kenya and estimate that the cost of fortifying maize through the upgrading of infrastructure, capacity of mills and training would be worthwhile and modest in comparison to upscaling the number of smaller mills (which are arguably much less efficient). In Zambia, where commercial use of hammer mills at the household level is high and market segmentation of maize products is well established (i.e., hammer, breakfast, roller maize meals) there may be scope of introducing fortification through improving the capacity of a subset of hammer mills in the country; though given the geographic locations and costs involved in training and quality assurance this is unlikely to be an option on a large-scale [36]. Assey et al. [37] have also shown, for instance, in Tanzania where support for small scale salt iodisation had resulted in support by way of machinery but much of these were not in use due to high running cost and lack of maintenance. To combat this, improving the efficiency of hand sprayers and knapsack-sprayers (commonly used at the small-scale) with the aid of supervision and post-testing improved the levels of salt iodisation to the required range of concentration. Large scale industrial milling can also cause technical complexities. Research has shown that industrial level milling can, for instance, cause iron to be physically lost during the milling of grain and this is exacerbated by the increasing amounts of imported processed cereals in LMICS [34,38]. Although this is less common in many high income countries (i.e., iron lost in milling) this may be an additional technological cost and thereby could be considered when fortifying another food vehicle e.g., “ultra” rice (fortified with micronized ferric pyrophosphate) which has shown promising results in reducing anaemia, particularly among young children [38,39]. 

**Figure 2:**
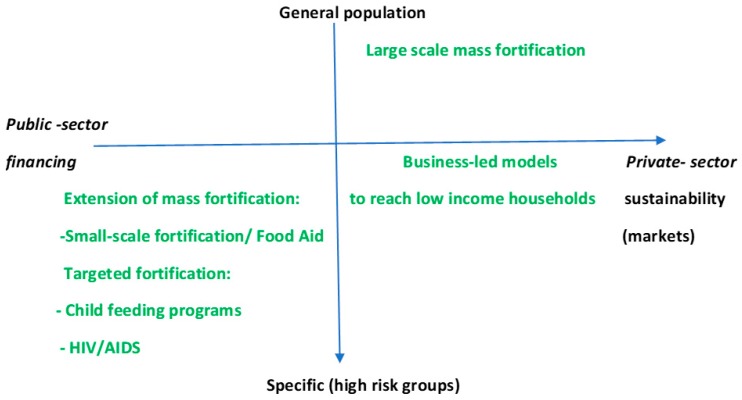
Schematic showing fortification delivery models. Source: Adapted from Hoogendoorn, Luthringer, Parvanta and Garrett [12], and Moench-Pfanner and Van Ameringen [43].

Different regulatory and legal frameworks and products have thus influenced the choice of vehicles used to deliver fortified produce to end users and achieve scale. A variety of delivery models have emerged (Figure 2). The vertical axis shows the degree to which the general population is targeted e.g., from specific (high-risk) groups/ targeted fortification efforts to the general population and larger scale mass fortification. Targeted groups may, for example, include individuals living with HIV that are supported with supplementary feeding programmes with fortified produce (e.g., whilst undergoing antiretroviral therapy) [40,41]. A probiotic fortified yogurt has recently shown promise in being “well tolerated” by people living with HIV in Tanzania (though no association was found with preserving immune function over the study period) this being said, the approach to developing the yogurt in a community-kitchen shows signs of promise for such grass roots nutrition-based approaches [42]. The horizontal axis shows the method i.e., financing i.e., public-sector financing to more business-led models [43] with functioning markets. There are cases where overlapping models exist (e.g., private-public/multi-sector partnerships) that may, for example, be a mass fortification effort but be publicly led and partly privately financed. Whilst some models are also aimed at targeted members of the population e.g., complementary foods for infants (aimed at undernourished), others can be promoted and considered a ‘premium’ brand and only be accessed by those with higher incomes [27]. 

Differences in fortification programmes and approaches have also been expressed regarding country landscape typologies and thereby influence the types of interventions (e.g., supply side or demand side interventions) [44]. This has a bearing on the particular bottlenecks experienced by private sector producers and public sector entities related to quality control/regulatory enforcement of quality standards. For example, Timmer [44] has hypothesised (with reference to universal salt iodisation (USI)) that four groupings exist with respect to level of regulation, infrastructure and coverage, namely:

(i) Countries with scaled-up programmes where optimal coverage exists but where there is a need to maintain oversight and ensure that disadvantaged/marginalised populations are reached.
(ii) Countries in scale-up phase, where high coverage has not been reached and engagement along the value chain (e.g., quality assurance and improvement of the quality/quantity of production (e.g., capacity of producers) is likely necessary as to will be improving coverage of particular geographies and market segments. Moreover, in this group there may also be need for a stronger focus on advocacy and awareness (communication efforts). Focus on the policy/regulation side will also be needed so as to control illegal flows of non-iodised/importation of non-iodised salt and presence of disincentives e.g., taxation. One challenge will be finding channels/strategies to reach disadvantaged people (e.g., subsidies or public distribution systems).
(iii) Countries without any policies or plans to scale up and achieve USI where there is a need to create more understanding and awareness among key gatekeepers (public, private, civic, academic sectors), about the function of USI to correct iodine deficiency, and to strengthen capacities to improve production and implement.
(iv) Fragile states where the particular enabling environment is not conducive to high coverage rates due in part to issues such as weak governance, political/civil strife, areas prone to natural disasters, etc. The relative importance and balance of formal and informal sectors seems to have received little attention in the literature. Given fortification strategies may be a low priority here, engagement of stakeholders will be necessary to try and redress this and improve capacity to implement salt iodisation. Temporary/alternate solutions in the meantime to improve coverage among target population groups will likely be necessary.

It should be noted that despite there being much success with mandatory legislation (particularly for staples) the need for quality assurance and monitoring is paramount in order to maintain consistency [12]. This includes standards being enforced and monitored for domestic importers of fortified produce as well as national level agents that are able to enforce necessary regulations. A recent assessment by the Food Fortification Initiative (FFI) in 2015 showed the scale of the problem highlighting that of 84 countries that had mandatory legislation at the time for cereals (such as wheat flour, maize, and/or rice) none had put in place appropriate monitoring tools or for the vast majority of countries no impact assessments had been carried out [12]. Table 2 shows the advantages and disadvantages of the various fortification approaches. 

### 3.2. Biofortification 

Biofortification is the process of increasing the density of vitamins/minerals in a given crop through agronomic practices (agrobiofortification) and/or conventional plant breeding, or transgenic breeding [13,45]. 

Fortification of plants using fertilizers rich in minerals has been practiced for centuries, for example with the addition of manure [46,47]. What is new is that modern agro-biofortification specifically targets nutrients of interest for human health [48,49]. Using conventional breeding, new biofortified varieties can be achieved by crossing cultivars that have dense micronutrient content (for the nutrient of interest) with cultivars with high agronomic performances [50]. When the gene coding for the specific micronutrient are not naturally present in the plant, genetic transformation is then an alternative strategy to enhance performance [45,50]. With genetic transformation, multi-nutrient trait biofortification (e.g., triple-fortified rice with β-carotene, iron, and zinc) was made technically easier [51,52].

Biofortification is a relatively recent approach compared to other nutrition strategies including fortification and supplementation. One of the first biofortification initiatives (transgenic as opposed to conventional plant breeding) was the Golden Rice Project. In the 1980s, the discovery of a genetic mechanism that made rice yellow and rich in β-carotene led to a large scale project launched in 2000 by IRR (International Rice Research Institute) and funded by the Rockefeller Foundation [53]. It was a failure at the time which was blamed on the lack of consideration of the local environment, low yield, and low acceptability due to public rejection of GMOs (genetically modified organisms). Today, genetic biofortification has still a long way to go because some of these issues remain unsolved (particularly related to regulatory/consumer acceptance of GMOs) [53]. In the late 1990s, Howard Bouis founded HarvestPlus—an interdisciplinary alliance of research institutions and implementing agencies in biofortification. HarvestPlus (HarvestPlus is one component of the CGIAR Research Program on Agriculture for Nutrition and Health (A4NH)) has invested heavily in iron, zinc and vitamin A biofortification since 2003 through conventional plant breeding. Staple crops are the preferred vehicle of biofortification because of their large consumption and lower cost compared to other commodities (Bouis, 2018, Personal Communication LSHTM). Other most commonly used micronutrients for biofortification are folate, selenium, and amino-acids tryptophan and lysine for high-protein maize [45]. There have also been attempts to genetically biofortify various crops with vitamin E [54], B6 [55], thiamine (B1) [56], and agronomically produce biofortified plants with iodine [57]. Goyer [58] argued that GM crops could bring a significant benefit in reducing the burden of micronutrient deficiencies and be economically viable. Biofortification was shown to be efficacious using conventional or GM breeding [51,52].

The main strategies employed by HarvestPlus are (i) rapid identification and adaptation of varieties which have significant impacts on micronutrients are “fast tracked” for release whilst other varieties that have a sole micronutrient focus have a longer lag-time to release; (ii) multi-locational regional trials across a wide range of countries to increase awareness of performance attributes and hasten release [13,59]. The HarvestPlus program now has eight focal countries (Bangladesh, DR Congo, India, Nigeria, Pakistan, Rwanda, Uganda, and Zambia). Cumulatively, more than 150 biofortified varieties of 10 crops have been released in 30 countries to date bred through conventional plant breeding rather than transgenic (genetically modified) which has faced stiff opposition with respect to food consumption [13,60].

The challenges of biofortification are to show effectiveness of the crops, refining delivery and marketing, and scaling up to integrate new crops into the food system according to [61].

Delivery models vary considerably between country and crop (e.g., for vegetatively propagated, self-pollinated, and hybrid crops). For example, a primarily commercial sector approach has been adopted in India and Zambia whilst various mixed public–private initiatives are found in other countries (Bangladesh, Nigeria, Rwanda, Uganda). Another model also includes an informal market systems approach which is primarily public (DR Congo) [13]. 

#### Timeline of Fortification and Biofortification 

**Figure 3:**
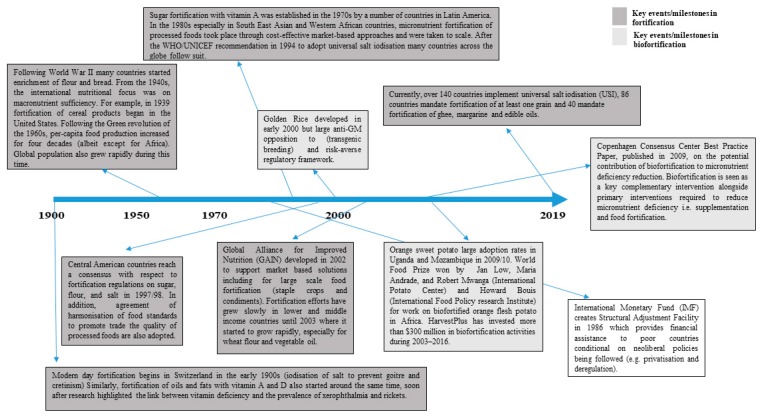
Timeline of key events in fortification, complementary foods, and biofortification Source: Adapted from [12,13,28,36,62,63].

Figure 3 shows some of the key milestones in fortification and biofortification fortification development. Very few players are involved in biofortification efforts worldwide with HarvestPlus considered the key/lead player [12]. 

The landscape of fortification is evolving with the development of global networks for nutrition such as GAIN that include nutrition as part of their core strategy. Fortification and biofortification efforts are increasingly part of the major world agendas for improving nutrition. 

### 3.3. Enabling Factors for Fortification and Biofortification Approaches

For fortification and biofortification initiatives to be taken to scale, there is a need to have an enabling legislative and regulatory environment to enable bodies to implement fortification and biofortification and encourage private sector engagement. In addition, the biofortified crops or fortified products require consumer acceptance as they have to be consumed in sufficient quantity and micronutrients have to be absorbable by the human body to have an impact on the nutritional status of people. Finally, the approach needs to be cost-effective in order for the business models to be successful.

#### 3.3.1. Acceptability

A major difference between fortification and biofortification has to do with acceptability. Whilst fortification uses food “vehicles” (e.g., flour, salt, oil) that people are accustomed to and therefore acceptance is not usually an issue (unless fortificants have a strong flavour or a different colour). Biofortified crops in contrast may either be of a different colour (e.g., orange-fleshed sweet potato rather than the white-fleshed one), taste, or may not exhibit similar agronomic properties (e.g., yield, appearance of the plant leaves) than conventional crops therefore an important component of the biofortification approach is promotion [64]. Perception of biofortified crops by farmers with regards to health and economic benefits are essential for adoption [65]. Measuring acceptability of biofortified crops is therefore essential [66]. This includes the development of appropriate marketing tools [66] to reach the populations at risk of micronutrient deficiency. Multiple attributes, including willingness to pay, have to be taken into account in the development of biofortified crops. 

Interestingly, farmers had a higher willingness to pay for biofortified crops when nutritional information was included [67]. Whilst many consumers of biofortified orange maize have experienced positive sensory benefits (e.g., taste) compared to conventional varieties, agronomic traits of biofortified cultivars seem to have been an impediment and thus need to be focussed in tandem with conveying the nutritional/sensory benefits [68].

Although some studies suggest that there is a major acceptance hurdle with acceptance of genetically modified biofortified crops, other studies have shown that: genetically modified crops did not generally encounter major reluctance from poor consumers as was the case in China for folate GM-rice and multi-micronutrient GM-rice (provitamin A, folate, and zinc) [51]. However, willingness to pay has been found to be higher with conventionally bred cassava (compared to GM-cassava according to [60,69]).

#### 3.3.2. Nutritional Impact

Whilst fortification strategies have proven effective because the micronutrients are added in their purest form to the food, with biofortification, the biology of the plants is involved and there is a need for proving the nutritional impact on the population of those crops [64]. In addition, biofortified crops are relatively novel compared to fortified products. There is now clear evidence that consumption of biofortified crops can improve micronutrient status of populations according to [51,52]. 

#### 3.3.3. Institutional and Regulatory Environment and Integration

The institutional and regulatory enabling environment are critical for fortification and for biofortification. According to [70], the enabling environment to improve nutrition should firstly support the improvement of the knowledge and evidence base, then improve policies and governance and finally improve capacity, partnership, and awareness. 

The integration of fortified products or biofortified crops needs to take into account the food system environment. Specifically, in the case of biofortification the involvement of smallholder farmers in the system is critical for its success given food processing is predominantly small scale and localised [71]. One of the reasons why GM-biofortified crops have shown limited examples of success is that regulation is stricter with GM-crops and has often not been implemented [72]. 

Furthermore, since the late 1980s and the introduction of structural adjustment programmes in many LMICs liberalisation of economies has occurred including privatisation of previously state own enterprise (nationalisation); a reduction in protectionist measures i.e., liberalisation of trade policies and altering public spending from heavy subsidisation to those aimed at broad-based economic growth i.e., education and infrastructure development. These liberalisation measures have had some positive impact, for example, the liberalisation of the seed market has enabled a plethora of maize varieties to be grown in Zambia and the mean lag time before release of a seed variety is far lower than in other countries in the region [73]. More recently, however, there has been a resurgence in protectionist policies that effectively crowd out commercial demand [73]. For example, input subsides towards fertiliser and maize create an artificial market for seed companies [73]. Thus efforts to commercialise biofortification in many countries including Zambia have also resulted in opposing views. Some have advocated an approach where one company is given exclusive rights in order to maintain brand image and purity whilst an opposing view is one which is centred around making germplasm freely available to all companies thereby engendering competition and long-term investment (a similar approach taken for white maize in Zambia) [73]. Issues such as these have also been raised regarding Ethiopia’s seed industry which has curtailed biofortification seed development and reach (Bouis, 2019, personal communication). 

#### 3.3.4. Cost-Effectiveness

According to [74] there are three types of costs in nutrition-orientated programs: (1) personnel (staff), (2) program-specific costs, and (3) capital. In fortification programs the fortificant (= micronutrient added to the food vehicle) is the major cost. Reference [75] reported that the fortificant cost represents about 77% of the total program costs on average but the cost may vary depending on the type, composition, and level of fortification. In addition, the cost of government in monitoring fortification (ensure that it is implemented over time) will vary depending on the capability of the government also the readiness of the government to implement fortification [75]. The other problem is that mass fortification may not reach the most vulnerable because they may not consume the food that is fortified [75]. This may be the case if the fortified product has an added cost. We hypothesised that could be the case, for example, if the product price has increased due to high value of the fortificant. The poorest part of the population may not be able to afford an increased price of their staple commodity. 

Cost effectiveness was estimated for a number of fortificants (iron, vitamin A, zinc) for fortification and supplementation. Fortification is overall more cost-effective than supplementation [76]. Iron fortification was proven to be cost-effective in Asia, Europe, America, and Africa [77] and was more financially attractive than iron supplementation because it required less resources. The cost per beneficiary per disability-adjusted life year (DALY) saved varied between $20–27 for Africa but was much higher for other parts of the world such as Europe. 

Cost-effectiveness has also been calculated for biofortified crops [11,78] and was around US$15–$20 per disability-adjusted life year (DALY) saved, which would constitute a highly cost effective strategy according to the World Bank [13]. Overall fortification and biofortification approaches have been found to be cost-effective and this is why they are part of the topic priorities quoted in the Copenhagen Consensus [2]. It is also necessary to compare the cost-effectiveness among ‘food vehicles’. For example, as a public health policy in some LMICS even though maize flour is heavily consumed; fortification may not be the most cost-effective intervention as previous analysis has argued that the most cost-effective interventions DALY saved, for Tanzania and Kenya, for instance, would be improving the scale of fortification of wheat [79]. 

The following section explores the strengths and weaknesses of these emerging models with reference to those used for fortification and biofortification

## 4. Types of Business Models Explored 

This section of the review considers the range of business models that have been reported in the literature and how they have been applied. We also consider whether there are measures of success and comparisons. We have organised business models into four different types: those that are public led, private led, multi-sector partnerships i.e., including international platforms such as GAIN or HarvestPlus, and community-led models. As with Figure 2, there is likely to be some overlap with respect to where certain models fit (e.g., public sector/private/multi sector models) which vary according to the different levels of engagement, country, “food vehicle”, and legislation.

### 4.1. Public Sector-Led

Public sector-led fortification programs have been successful in a number of countries (see Figure S1) largely as a result of mandatory fortification (legislation), enforceable regulation and a mixture of incentives [80]. For example, the Philippines and Costa Rica have had successful fortification experiences with fortified rice. This includes the development of legislation and market incentives [81]. Despite the fact that the engagement of the private sector is vital, it is argued this on its own is not enough and governments often need to take a stronger role (in lieu of a strong private sector) and bear some of the risk [81]. For example, in the Philippines, financial burden to develop markets has solely been with the public sector, whilst in Costa Rica there has been public and private financing resulting in faster progress than in the Philippines. Quality assurance and compliance has been key to success as has been generating consumer demand. Costa Rica has benefited from “universal coverage” which has negated the need to generate demand; whereas the Philippines has had to rely on the private sector commercial marketing [81]. In Uzbekistan, national fortification programmes were supported by targeted social marketing to the media, millers, and bakeries- mandated packaging was also established on flour packaging [82]. Also in Uzbekistan, the government subsidised laboratory equipment and in order to improve compliance laboratories were required to submit a business plan to ensure equipment would be used to enhance food quality testing across the board and not solely related to fortification [82]. 

In LMICs, some of these failures are more apparent and national fortification strategies have had varying degrees of success compared to developed nations. The reasons for failure are varied and can vary from one country to another, by product, labelling/branding and whether the fortification is mandatory or voluntary. For example, a recent study of mandatory fortification of vegetable oil, sugar, and cereal flours in Nigeria found very few retail outlets were compliant i.e., between 60 and 90 percent of products fail to meet the required fortification standard largely because of the inadequate fortification premix at an industrial level or technological/other constraints that impacted the quality of the premixes. Ineffective monitoring of compliance by the regulators, and self-regulation was also a contributing factor [83]. In Tanzania, mandatory fortification for maize flour has been introduced, but given consumer habits (e.g., maize purchased in informal markets) this has not only impacted on reach i.e., reaching vulnerable members of the population but also hampered buy-in from private sector flour mills [84]. As in Tanzania, the substitution (This term is commonly used in economics to refer to the change in demand which might take place if, for example, a price of a good increases relative to its substitute; consumers would likely switch to consuming the next best (cheaper) alternative) effect may also limit success of such programmes especially if fortified products come with a higher price further limiting their coverage [84]. A wide-ranging review of eight countries with a number of mandatory fortification strategies also found the lack of impact is due to the inability to generate access, program design failures and lack of monitoring, evaluation, and learning related to implementation efforts [85]. Incorporating fortification efforts into social safety nets has also been piloted through “targeted fortification” [86]. In India, GAIN, supported the government by providing the provision of technical and financial assistance at different stages and helped to create a partnership between the private sector (Gujarat Roller Flour Millers Association (GRFMA)) and the state government. The state government then agreed to follow voluntary fortification (which allowed consumers to choose to purchase fortified or unfortified produce) and provided branding with a logo for fortified produce. The government then agreed to swap wheat grain for wheat flour in its social safety net programmes (SSNPs). This provided an opportunity for a large “new” market for many of the larger millers in the state. An “open market test” (pilot test) through selling the product through bakeries and hoteliers also enabled the government to examine the potential risk before committing resources. The Gujarat experience led to increase in uptake of fortified flour and a reduction in micronutrient deficiency among beneficiaries of the SSNPs. In addition, the initiative also led to a growth in the market share of formal sector millers though informal market saw a reduction in market share [86].

### 4.2. Private-Sector Models

Private-sector models of fortification follow voluntary fortification which invariably focus on commercial market development and may have varying degrees of input from the public-sector involvement. An example recently from Brazil focussed on key areas including (1) expanding production of fortified kernels of rice; (2) building a supply chain through commercial rice mills; and (3) generating demand through social marketing. Despite an increase in awareness of the product the relevance of the product among consumers was low. Support from the public sector particularly through incorporating fortified rice into nutrition/safety net policies and programs is likely needed to build trust and consumer confidence [80]. There have been examples, however, where voluntary fortification has been used/piloted at the regional and local scale and then used to influence government to mandating legislative fortification programmes (e.g., vegetable oil in India and flour fortification in Kenya though sustainability of this intervention was unsure) [12]. Voluntary fortification or market driven whereby a food manufacturer takes a profit driven approach have rarely gone to scale in LMICs [87]. Agnew and Henson [88] have shown the challenges of bringing a fortified yogurt to scale in Bangladesh; affordability being the main reason that consumers did not purchase the product; however, challenges in marketing (particularly in facilitating repeat purchasers) and distribution were also cited as significant challenges. Market driven approaches such as the case of Tiger biscuits (aimed at poorer households) in India have also been unsuccessful in reaching scale; due in part to low awareness among poor consumers, cheap alternative foods, and a unfavourable institutional environment e.g., lack of food safety standards/regulation [89]. 

### 4.3. Multi-Sector Partnerships 

It is clear that whether public-led or private sector initiated, partnerships and buy-in across the board are needed to reach scale or reach specific members of society. In recent years National Fortification Alliances (NFAs) have emerged (e.g., Senagal, Tanzania). These approaches consist of public sector bodies, the private sector and civil society organisations. Hoogendoorn, Luthringer, Parvanta, and Garrett [12] highlight common partners in a NFA include government actors which should include: (i) a lead ministry, (e.g., Ministry of Health or the Ministry of Industry; and (ii) national or provincial/county food control authorities that are responsible for food safety. Private sector involvement should include actors related to the food processing industry and can include for example, millers, refiners, and suppliers/manufacturers of vitamins/minerals. Finally, other players can include retail organisations, academia, and consumer organisations which will support the social marketing/information delivery to end users. Others have noted that NFAs have been critical to supporting fortification efforts and play a role of oversight and guidance in order to improve fortification programs [87]. In India, an example of a successful state food fortification alliance in Madhya Pradesh which engaged all relevant stakeholders has led to edible oil, wheat flour, and milk being fortified on mass scale annually and successfully marketed [90]. Similarly, other multi-sector coordination mechanisms exist which usually focus around a specific micronutrient (i.e., iodine or Vitamin A coalitions) such as through the SUN (scaling up nutrition) movement/business networks [12]. It should be noted, however, that a number of national fortification initiatives often do not reach very remote communities and fail to reach scale because of inadequate funding mechanisms [91]. 

The biofortification approach, in particular that promoted by HarvestPlus in the last 10 years, has been mostly a multi-sector partnership [92]. Since biofortification depends on seed and, to some extent, producer own-consumption of the improved crop, the debate on business models is strongly related to the literature on food self-sufficiency and models for extending improve farming methods, commonly termed “extension”. Therefore, differing views on extension approaches for biofortification have also been explored as these are proxies for business models. De Groote, Gunaratna, Fisher, Kebebe, Mmbando, and Friesen [68] found that the classical approach to encouraging farmers to take up improved plant varieties and include them in their farm business model which focussed on the agronomic traits was more successful than a pure focus on the nutritional benefits of biofortified crop consumption as a business model.

As with fortification programmes, multi-stakeholder platforms/partnerships have shown promise. Modes of delivery differ significantly from country and by crop type. For example, in Zambia given the dominance of hybrid seed companies biofortified crops were solely licensed to these companies for commercialisation and development. In contrast, in Rwanda HarvestPlus used a different model by focussing on contracting farmers to produce/multiply seed and by engaging cooperatives, and small seed companies [13]. Vaiknoras et al. [93] analysed two models of delivery in Rwanda for iron-biofortified beans. These were direct marketing, i.e., contracting seed multipliers to produce certified seed and farmers are able to then purchase this seed through authorised agro-dealers and the second being payback (or more recently termed “seed swap”) whereby farmers would payback a portion of their produce to HarvestPlus in exchange for seed. The study found that whilst direct marketing was able to have higher reach and increase initial adoption; farmers then struggled with access to subsequent seed and villages which had initiated payback disadopted at a lower rate than villages where payback was not present. Significant scope exists for upscaling vegetatively propagated crops (stems, tubers, or vines rather than seeds). Seed systems are often small scale rather than commercial due in part to the perishability and expense in transport costs [13]. Three critical elements have been identified as imperative to ensure the success of ‘upscaling’: (i) supply related i.e., engagement of research, public and private sector bodies to commit to varietal release/development including the National Agricultural Research Systems (NARS); (ii) policy level champions that recognise the importance of biofortification to public health; and, (3) demand driven support to rural and urban consumers to start demanding content in their staple crops/foods [13]. Agronomic challenges have also been cited as challenges to be addressed. For example, cultivating these new varieties may take more time than traditional varieties (e.g., weeding time is longer for orange-fleshed sweet potato growers in Mozambique compared to traditional varieties) so along with access to varieties farmers may need adequate training and support to be able to produce such crops [94]. 

The challenge with biofortification is that it requires the development of a new value chain for the crop—from the seed system to the marketing of the crop—and ensure the integration of the different public and private actors for the sustainability of the delivery of the crops.

### 4.4. Community-Based Models 

Community based model interventions have been trialled successfully in a number of countries. These are usually supported by NGOs. For example, WorldVision supported efforts for staple crop fortification in Malawi, Tanzania, and Senegal through a mixture of support for medium scale units (e.g., to support the purchase of premix for small scale/home fortification units; e.g., purchasing in bulk and subsiding pre-blend sales to small scale producers which did not incur the added costs of having to test their produce at local mills to make sure requirements were met) [91]. These activities through support of NGOs are usually coupled with complementary development activities. In Senegal, components of the project included building two mills in the community to process grain, which offered the community an alternative to the labourious method of milling. These were run as “community managed” businesses with profits being kept by the operators [91]. Home fortification then took place as households used a pre-blend to fortify the pounded grain supplied by the mill. In all cases however, the projects ceased and were not able to achieve cost recovery when funding was ended. This type of fortification is often also termed point-of use fortification [87] and has been shown to be successful in a number of contexts, for example, in Vietnam [95]. Andrade et al. [96] has also shown the successful development and acceptance of point-of use fortification among school-aged children in Honduras; highlighting the addition of micronutrient powder to traditional dough making which produced noodles that are mixed in with customary rice meal. Another example of home fortification is BRAC’s recent initiative in Bangladesh which used frontline community health workers to promote use of micronutrient powders with better feeding practices [97]. Whilst the sustainability and scale out of these models are not clear, targeted programs such as these are likely to hold promise. For example, the case of home fortification in Vietnam (micronutrient powders to caregivers) has been successfully converted from a pilot project to being approved by the Ministry of Health through a formative policy change and formed part of their initiatives to combat micronutrient deficiency nationwide [95]. 

### 4.5. Levels of Business Models for Fortification and Biofortification

The literature reports various levels of complexity in business models ranging from public to public–private partnerships (with engagement of other stakeholders). These are also related to the level of commitment of the private sector to sustainable development [98]. Four levels are reported ranging from an individual company to a multi-stakeholder institution. Level 1 relates to cooperation with business partners along value chains (this involves minimum involvement from the private sector to sustainable development including nutrition). Level 2 is situated at the project-level i.e., financing and implementation of partnerships; small scale companies linking with other bodies such as investors, governments, philanthropic organisations, NGOs, and research centres. Level 3 is industry-level precompetitive business alliances: association of companies: industries and corporate bodies (this is a more organised approach towards sustainable development). Finally, level 4 consists of multi-stakeholder institution platforms and networks: those can be formal or informal platforms but with a high level of commitment to sustainable goals and development. These can be complex, such as groups or companies collaborating with donors, investors, and academics. One example is GAIN (Global Alliance for Improved Nutrition) [98]. GAIN was funded as a result of a United Nations (UN) initiative in 2002 as a non-profit foundation. It is a platform that helps various nutrition actors—governments, UN agencies, non-governmental organizations, and businesses—to build collaboration in order to tackle malnutrition. 

GAIN has an output-orientated model which encourages partnership at different levels but arguably does not tackle the roots of malnutrition [99]. Other output orientated actions are the Iodine Network and the Food Fortification Initiative (FFI). On the other hand, the International Alliance Against Hunger (IAAH), an initiative from IFAD (International Fund for Agriculture and Development), is a process orientated model that seeks to develop strategies with “stakeholders advocating a common cause” and a “process-oriented partnerships tackle broader problems with higher democratic standards, but less business attention” and a bottom-up approach ([99] p. 4). In practice, GAIN’s approach involving the private sector has received much attention [99]. The authors argued that considerable business involvement and encouraging partnership in an output-orientated approach is more efficient although the risk is that the poorest economies may not be included because businesses would rather support emerging economies at the risk of neglecting the least developed countries. 

## 5. Discussion and Conclusions

### 5.1. Analysis of Key Themes, Gaps, Approaches, and Lessons in the Literature

This review seeks to bring together the information about the approaches used to fortification and the various business models that have been reported. We have also sought to highlight gaps in the available information. Table S4 includes 30 of the key articles identified from this review that have explored particular gaps/challenges or assessed business models relating to the fortification of staple foods/complementary foods and in a wide variety of countries/regions in LMICs. From the list reviewed, the vast majority of articles/specific references to large-scale mandatory fortification focus on wheat, maize, or oil (eight references whilst there are very few on other staples e.g., two on rice and one on sugar). The majority of these are examples from Africa (including North Africa, West Africa/Eastern Africa Morocco), East Asia (Vietnam and Indonesia), and Central Asia (Uzbekistan) whilst three examples of rice fortification are from the Philippines, Brazil, and Costa Rica. 

The most frequent gaps/lessons identified for large scale fortification/mass fortification efforts to be effective are the choice of food vehicle alongside access to the specific premix (or the adequate addition of fortificant)/necessary fortification equipment. 

The importance of strong legislation which includes quality assurance and compliance monitoring [81] and failure to reach poorer segments of society due to price (e.g., wheat flour and oil in the Philippines) are key lessons. Funding and sustainability is also identified as a key constraint to success, scale, and sustainability of business models. The advantage of centralised production with a few players is seen as a key to success (See Table S4). 

Primary bottlenecks for private sector and importers (across scale) are premix costs equipment running costs. For example, import duties/taxes on premixes or fortification equipment can result in higher prices for fortified produce and can deter the private sector from investing [35].

The legal framework within which the business model resides is clearly crucial and we have found that the mandatory/voluntary debate is among the richest in the literature. For example, Indonesia recently had issues with a change of fortification strategy from a highly successful mandatory fortification of flour to opting to change this to voluntary because of pressure from flour importers arguing that mandatory fortification was a barrier to trade which led to a decrease in uptake of fortification by producers. This decision was eventually reversed after it was shown that mandatory legislation was not affecting flour imports or flour prices [100]. The legal framework is thus described as imperative as this has a bearing on implementation and should include the food vehicle being fortified, micronutrients specifications/procedures, and responsibility of fortification (mandatory or voluntary) including procedures for quality control and compliance (ensuring that capacity is there as this has severely hampered efforts even when this has been written into legal frameworks). Adding social marketing and social business models into this mix has largely unknown consequences and is a gap in knowledge at present. Nelson [100] highlights that clear costing and methods for social marketing should also be stipulated.

Rice has been found to be a particularly problematic staple to introduce through voluntary/mandatory means due to the cost of the coating technology (Columbia), cultural acceptance/low coverage (Philippines) and low coverage rates in Brazil (See Table S4). These are unlikely to be successful on a large scale without mandatory legislation and strong enforcement (e.g., in the case of Costa Rica) including the product being similar to fortified produce on the market. Successful cases where voluntary fortification have been successful have been found in India where the produce is integrated into Social safety nets and through engagement/incentives given to associations, e.g., wheat flour [86]. In this case strong public sector commitment is needed. Small-scale fortification challenges are also dependent on the staple. For rice challenges were one of social marketing as fortified rice is a niche market with very little coverage in Brazil. Though overall household coverage was higher with voluntary fortification the spray technology employed has not been tested and thus the efficacy of fortification has been questioned [30]. Millers in Columbia were also unwilling to switch to more proven methods because of the costs involved (i.e., extrusion or coating) [30]. Moreover, the enforcement standards for domestic and imported rice is also critical to ensure consumers benefit from rice fortification efforts [30].

At the smaller/medium scale, the examples of other staple foods relate mainly to salt fortification and maize/wheat fortification also focussed on Africa (mainly Eastern Africa i.e., two references; one reference to East Asia Vietnam and Indonesia and one based in India—see Table S4). The case of salt is somewhat similar to rice though low-cost/low-technology fortification strategies have been developed which improve the fortification levels of the required micronutrient in the case of Tanzania they have been less fruitful in other countries, e.g., Tanzania and Mozambique [91]. Issues with respect to enforcement, fragmented production (mainly small-scale producers) and legislation loopholes that allow iodised salt to be shipped and traded (to be fortified elsewhere) also inhibit fortification efforts [100]. For smaller scale maize flour fortification, the technology/equipment is a significant barrier as is the premix supply. Large-scale production units are able to gain from economies of scale related to fortification, for example: marketing/distribution and quality control, that minimize the additional cost of fortification to consumers. In contrast, small-scale village hammer mills, for instance, struggle with handling the cost of the premix, transport/storage to often very remote locations and additional quality control measures which significantly reduce viability [91].

Thus, the need for multisector partnerships that ensure oversight and the engagement of a variety of stakeholders is seen imperative especially for large-scale staple fortification efforts be this voluntary/mandatory led. Small-scale initiatives would also be enhanced if these are then brought into national fortification frameworks, e.g., salt [91]. Though it should be noted that even when these have been integrated into mandatory legislation efforts National fortification initiatives have often failed to reach very remote communities [91].

This review also identified small/medium-scale initiatives for non-staples that are voluntary/non-specified interventions (e.g., micronutrient powders or other products (biscuits, yogurt)). These include examples found in Central America (Honduras), South Asia (Bangladesh), Eastern Asia (Vietnam). Small/medium-scale initiatives are also hampered by a need for training/maintenance and the high running costs of fortification is a key challenge (i.e., sustainability). A case where voluntary fortification has been successful on a large scale is the example of condiments/seasonings in South East Asia which has benefited primarily where there are centralised production, i.e., few firms with high market share. Social marketing/awareness among populations is a key constraint challenge in other situations identified [88] alongside distribution challenges/reaching the poorest due to price and an appropriate regulatory environment [89]. A positive example here could be the tiered price system for a locally produced micronutrient powder in Vietnam; marketed to caregivers of infants based on their disposable income which showed a positive correlation between the number of sachets bought by caregivers and the wealth index [95]. For example, poorer households purchased single sachets and packets of 10 whereas wealthier caregivers preferred the larger boxes with 60 sachets [95].

Table S5 shows the handful of publications on biofortification with relation to business models/assessment. The focus of these is primarily on South Asia and Eastern Africa. Here, models have explored public–private initiatives, but delivery models are in reality large-scale initiatives. Public-sector led models do exist, e.g., DC Congo [13]. Where seeds have been successfully released, buy-in from seed companies with an established market has been critical. Options for smaller-scale village multiplication and dissemination through social networks have been successful in Rwanda [93]. Thus, a key lesson has been one of sustainability i.e., not having the adequate amount of quality planting material at the local level after initial adoption takes place [93].

A key gap (also in most of the cases) across scale and legislation (e.g., mandatory or voluntary) or fortification type/ product (biofortified/fortified staple/complementary food) e.g., is the lack of reference to the business model particular, prior to program implementation and program implementation while cited as key to sustainability [35]. For example, better diagnostic work is needed in order to identify where fortification/biofortification is likely to have an impact (e.g., where the product has already very high coverage rather than divesting resources where impact is unlikely [12].

Though voluntary fortification models have been largely unsuccessful in LMICs, options for small, localised communities or home fortification may be beneficial, particularly in remote rural settings. Clearly, whether programmes are designed for fortification or biofortification multi-sector partnerships/platforms are likely to be needed to reach scale and ensure oversight. However, multi-sector platforms can also fail to reach the target and require innovative methods including realistic funding mechanisms [91]. Similarly, biofortification models have also been successful with multi-level partnerships but where regulatory/institutional environments have been challenging (e.g., unfavourable seed systems; research and development infrastructure) difficulties have occurred [13].

Overall, particular gaps exist in LMICs to support training of professionals (vocational or tertiary level education) in food safety/equipment which in turn will strengthen existing public-sector bodies. Likewise, it will be necessary to support and train extensionists using methods such as Farmer Field School [101,102] or Innovation Platforms [103] and support curriculum development in academia (e.g., biofortification/biofortified crops to be included under agricultural sciences/agronomy). As with fortification efforts, elements of social marketing may also be needed in order to encourage the use/consumption of new varieties [12]. Social marketing may also enable a better understand of tastes/traits consumers prefer which may influence the choice of food vehicle to be fortified or biofortified crops/practices being produced [80,86,88]. Having said this, the literature is relatively silent on the subject of who might finance this effort. Most countries have publicly funded free extension services that perform poorly. Success has come from new models of farmer led extension, but costs remain high and coverage poor. The capture of extension service benefit by the private sector, less isolated, less vulnerable, and the balance of public/private, paid for (toll)/non paid for and ability to exclude others from benefiting vs. the spill-over benefits from common goods is a consistent element of the discourse going back some time.

New mobile phone bases systems show promise in addressing these issues, particularly to reduce transaction costs, but are still immature and widely unproven [104].

### 5.2. Towards a Conceptual Framework for Food Fortification Business Models

**Figure 4:**
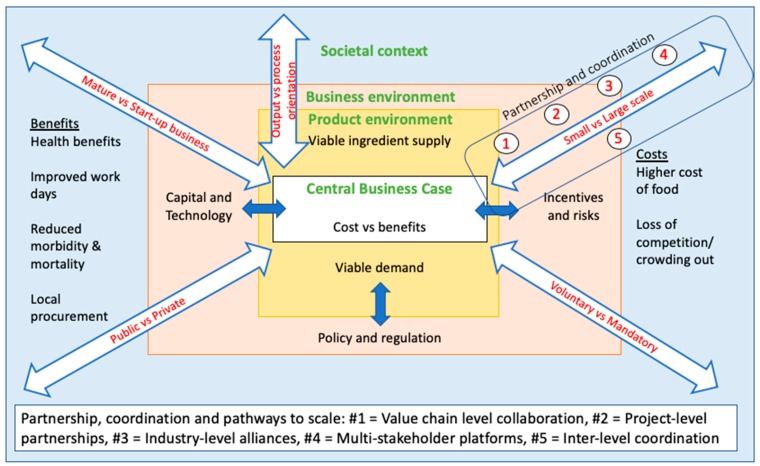
Initial structure for understanding different business cases.

Using the literature reviewed and the range of parameters and models that have emerged, we posit an initial structure against which practice (e.g., project) can be assessed and typified (Figure 4).

Taking a business orientation, viability is core to every aspect of the model. Failure of income to exceed costs results in firm failure. This “Central Business Case” underpins all food fortification businesses to a lessor or greater degree depending on the amount of subsidy provided by the public sector. This subsidy comes in many forms and for many reasons: to promote start-up and early adoption, to overcome scaling and investment challenges, to address aspects of market failure, and to respond to decisions and needs in the wider society. Sometimes this choice of business model may simply be related to the agenda of whoever is promoting the particular fortification initiative—not all economies are able to impose agency over domestic food policy, especially where the state is fragile. Each business format or model is in itself located within a “product environment”. For example, fortified cereals compete with substitutes in the same market space, so it is hypothesised that, to compete in this product environment the same business case needs to be made with viability of supply and demand assured for business success. Expanding further, each fortification solutions or proposition needs sufficient funding (capital), appropriate technology, a suitable regulatory environment, and supporting policies [105,106]. At this level, the role that incentives play in managing business risk seems to be important. Finally, fortification programmes fit into a wider societal context where decisions have to be made between investments that bring about societal benefits and externalities. This realm pits health benefits and greater work effort from food fortification against, for example the distorting effects that subsidies have on the wider economy, resulting in economic inefficiency and sub-optimal allocation of resources. One caveat is that not all fortification initiatives have the same starting point. Most, it seems, begin as a public sector, donor led, non-government organisation supported initiative; a trial or a pilot [86]. Scale, impact, and sustainability (e.g., cost recovery) drive these initiatives toward varying elements of private sector engagement, with varying degrees of success, so the elements of “public”, “private”, “voluntary” and “mandatory” in Figure 4 are interchangeable. The range between process and output orientations is expressed by the link between the central business case and societal context: processes being driven by stakeholders in society and outputs by more economic imperatives.

### 5.3. Conclusions

The following are conclusions and recommendations on improved/optimised private sector business models to sustain production and marketing of quality fortified foods, and mechanisms for public sector oversight in assessing effective population coverage of fortified/biofortified foods.

Despite the huge literature available on fortification, we find that the discourse on business models and their success or failure to deliver the hoped-for benefits of different kinds of food fortification is somewhat limited. While large data sets exist that share information on health aspects and coverage [85], the viability of each initiative is seldom considered.

Much of the available literature reports apparent success [26,82,86]. Failure is under-reported with few examples [30,81,91]. We find that some commodities are considered by more literature than others, for example wheat flour, salt, and oil is widely reported and to some extent maize flour, but sugar and rice fortification has received less attention [26]. Recent efforts at biofortification have been high profile with global coverage. This may leave a distorted impression of the relative cost benefit of biofortification vs. fortification.

### 5.4. Gaps and Missing Issues

- Models of extension approaches and seed multiplication delivery where state involvement in seed systems is still very high and can ‘crowd out’ private sector research and development need further exploration.
- Focus on methodologies that will support the focus on developing positive agronomic traits as this is still a key area of concern in biofortification as to are appropriate extension/delivery models [93].
- Biofortification-scope exists for upscaling vegetatively propagated crops (e.g., stems, tubers, and rice) [13].
- Increasing fortification efforts in rice since this is still relatively low [26,38,39]. Similarly, low rates of maize flour fortification in countries which are highly dependent on maize may provide potential for fortification programmes (including where relevant small-scale fortification) or support for small-scale fortification [37].
- Better diagnosis of the specific value chain where interventions are proposed [107] and including where biofortification and fortification is most needed/will be most beneficial [12]. This could also help identify where ‘targeted’ fortification, e.g., through community level development/home fortification programmes are likely to have the most impact.
- Whilst more needs to be done to make biofortifed crops agronomically appealing; social marketing is still a key issue for fortification and biofortification.

### 5.5. Bottlenecks

On the basis of the literature reviewed we hypothsise that the main bottlenecks to successful delivery of food fortification business models are:

- Viable supply of suitable ingredients, including, where appropriate import of equipment and ingredients;
- Access to capital and technology to start and grow suitable businesses;
- Suitable incentives to mitigate the risks, particularly of early adopters of new technologies; and,
- Unconstrained and viable demand for the fortified/biofortified product.

### 5.6. Key Drivers

With respect to the key drivers we hypothesise that the key drivers to successful delivery of food fortification business models are:

- Scale of operation, with larger being easier than small scale businesses;
- Business maturity at the initial point (e.g., start-up vs. mature);
- Both public and private sector engagement;
- Mandatory inclusion and governance (particularly for fortification);
- The tradability of the key factors: technology and ingredients

In our initial structure, we place these drivers and bottlenecks identified in the literature within four key contexts: the central business case (e.g., the individual fortification or biofortification business); the product environment (e.g., the market for the specific flour or biofortified food); the business realm (e.g., the conditions that make all businesses in a given economy flourish or fail); and the societal context (e.g., the wider benefits or costs to society as a whole and the possibility that individuals, firms, and governments can capture these benefits).

### 5.7. Limitations

This review has limits. The method (e.g., using key words) may have missed some important references, particularly where these are not in English. The literature may have a bias towards reporting interventions where there has been an associated research effort. Many interventions have no related literature, so aggregation from this data is inherently risky in that it will tend to highlight success and the highly researched elements for fortification.

### 5.8. Recommendations

We recommend that, in order to address the research short-falls, in depth case studies should be conducted and wide spread expert interviews are completed using a formative approach [108]. However, the initial structure put forward to understand different business cases for food fortification and biofortification would point us towards the following generalisations:

It is easier to work with a small number of larger businesses than a large number of smaller ones and, therefore, in order to achieve the wider societal benefits, supporters of food fortification should not be afraid to adopt a “start at scale” approach. However, policies to protect the vulnerable and to prevent anti-competitive or crowding out practices are needed.

Where scale is achieved by large numbers of smaller businesses (and also by small number of larger businesses), then coordination and partnership is crucial for success. The evidence seems to suggest that this is less important where mature, large scale businesses already exist.

Almost all texts point to the greater success of projects where fortification is mandatory and adequately monitored and regulated. Supporting project with more regulation seems to yield greater success, so this highlights the strength of fortification efforts based on mandatory inclusions.

Projects dependent upon a high level of tradeables (e.g., imported ingredients or technology) are more prone to failure and therefore domestic ingredients and technology should be preferred.

A balance of public and private sector engagement is important, but not always essential (e.g., where horitzontal coordination is strong in a given sector). We would recommend that this balance is contextual to the commodity, sector, and country.

Mature businesses (e.g., well established, large, grain millers) are more likely to implement a business model because they are experienced and much of their capital investment is already sunk. For rapid uptake, working with mature businesses is recommended.

Developing viable business models takes time and is complex. Most texts recommend that, as well as measuring absolute outputs, interventions should set targets for key elements of the business development process.

## Figures and Tables

**Table 1:** Theme and terms used in the literature search.

Search Theme	Search Terms	Search Results
Fortification	“Fortification” AND (“Business” OR “market” OR “multinational “OR “private sector” OR “trade” OR “enterprise” OR “economics” OR “cost effectiveness” OR “scale”)	9457 articles found. After removing duplicates and non-related articles = 89. Relevant articles selected = 39. Full text available = 36. Additional texts (pearl search) = 21.
Complementary foods	“Complementary foods” AND (“Business” OR “market “OR “multinational “OR “private sector” OR “trade” OR “enterprise” OR “economics” OR “cost effectiveness” OR “scale”)	756 articles found. After removing duplicates and non-related articles = 386. Relevant articles selected = 18. Full texts available = 17. Additional texts (pearl search) = 2.
Biofortification	“Biofortification” AND (“Business” OR “market “OR “multinational “OR “private sector” OR “trade” OR “enterprise” OR “economics” OR “cost effectiveness” OR “scale”)	1684 articles found. After removing duplicates and non-related articles = 610. Relevant articles selected = 31. Full texts available = 27. Additional texts (pearl search) = 30.

**Table 2:** Pros (+) and cons (−) of fortification approaches by vehicle, scale, and legislation.

Vehicle for Fortification	Large-Scale Mandatory Fortification	Large-Scale Voluntary Fortification	Small-Scale (voluntary/mandatory)
Wheat	Centralised production/few players e.g., Egypt (+) Economies of scale (+) Very few have implemented appropriate monitoring tools (−) Lack of impact assessments (−)	Unable to reach the poor due to high price (−) e.g., Philippines Scale-only a small proportion of industrially milled wheat is voluntary fortified (−)	Nb: few examples found
Maize	Cost of fortifying through large scale mills (in eastern Africa) is seen as cost efficient rather than focus on upgrading smaller mills (+). Lack of impact assessments (−)	Nb: few examples found	Lack of adequate mills to fortify maize (−). High dependency on small-grain mills particularly in Eastern Africa (−). High consumption of unfortified maize at the local/household level (−).
Rice	Very few have implemented appropriate monitoring tools (−) Lack of impact assessments (−) Voluntary or mandatory fortification of rice with iron is still uncommon (−)		“Ultra” rice ^1^(fortified with micronized ferric pyrophosphate) which has shown promising results in reducing anaemia (+) Taste and preference for non-fortified produce in some countries e.g., Philippines (−)
Salt	High coverage rates in recent years for countries with mandatory fortification (+)	Modest increase in household coverage for countries following voluntary fortification (−)	Options such as improving the efficiency of hand sprayers and knapsack-sprayers with the aid of supervision and post testing can improve improved the levels of salt iodisation (+)
Yogurt		Failure to reach scale (−)	
Oils		Unable to reach the poor due to high price (-) e.g., Philippines.	
Sauces		Philippines and Cambodia success with sauces due to centralised production/ high market share (+)	

^1^ The technology is scale dependent. We have included it under small-scale as it is a relatively low-cost/simple method for addressing iron/micronutrient deficiency.

## Supplementary Materials

The following are available online at https://www.mdpi.com/2072-6643/11/7/1594/s1, Figure S1: Summary of number of voluntary and mandatory fortification programs in different parts of the world, Figure S2: Summary of number of voluntary and mandatory fortification programs for the various food vehicles, Table S1: High income and upper middle income countries by regulation type, food vehicle and household coverage/proportion industrially processed, Table S2: Lower middle income by regulation type, food vehicle and household coverage/proportion industrially processed, Table S3: Low income countries by regulation type, food vehicle and household coverage/proportion industrially processed, Table S4: Review of key knowledge gaps/policies/ business models by vehicle/region and scale, Table S5: Biofortification review of key knowledge gaps/policies/business models.
