# Supplementary material for: Which Choice of Delivery Model(s) Works Best to Deliver Fortified Foods?
Source: Nutrients. 2019 Jul 14;11(7):1594. doi: 10.3390/nu11071594 (PMC6683040; doi:10.3390/nu11071594)
Supplement: Supplementary file 1 [file nutrients-11-01594-s001.pdf]

Figure S1. Summary of number of voluntary and mandatory fortification programs in different parts of the world.

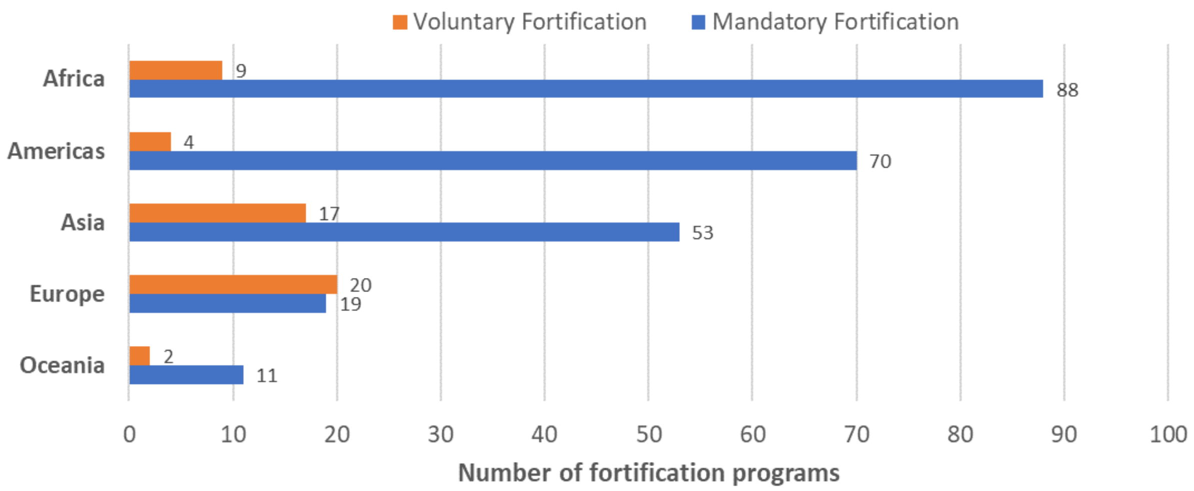

Figure S2. Summary of number of voluntary and mandatory fortification programs for the various food vehicles.

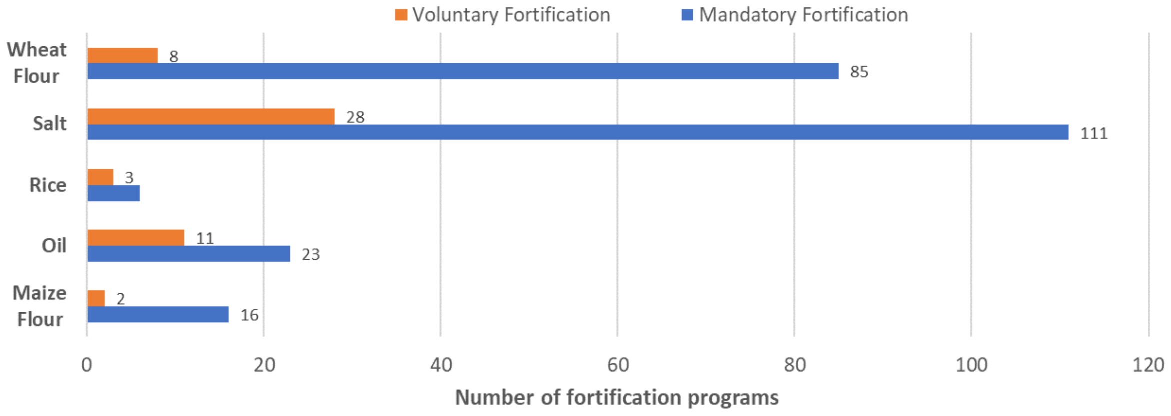

Table S1. High income and Upper middle income countries by regulation type, food vehicle and household coverage/proportion industrially processed.

Country	Year	Food Vehicle	Indicator	Income Status	Region	Coverage of industrially produced, fortified /total population (%)	Proportion industrially processed (%)
Albania	2008	Salt	Mandatory Fortification	Upper Middle Income	Europe	91	NA
Algeria	1990	Salt	Mandatory Fortification	Upper Middle Income	Africa	81	NA
Antigua and Barbuda	1992	Wheat Flour	Mandatory Fortification	High Income	Americas	NA	NA
Argentina	1967	Salt	Mandatory Fortification	Upper Middle Income	Americas	NA	NA
Argentina	2002	Wheat Flour	Mandatory Fortification	Upper Middle Income	Americas	NA	NA
Australia	2015	Salt	Mandatory Fortification	High Income	Oceania	NA	NA
Australia	2014	Wheat Flour	Mandatory Fortification	High Income	Oceania	NA	NA
Australia	2017	Oil	Voluntary Fortification	High Income	Oceania	NA	NA
Austria	1999	Salt	Mandatory Fortification	High Income	Europe	NA	NA
Azerbaijan	2001	Salt	Mandatory Fortification	Upper Middle Income	Asia	93	NA
Bahamas	1992	Wheat Flour	Mandatory Fortification	High Income	Americas	NA	NA
Bahrain	2012	Salt	Mandatory Fortification	High Income	Asia	NA	NA
Bahrain	2002	Wheat Flour	Mandatory Fortification	High Income	Asia	NA	100
Barbados	1992	Wheat Flour	Mandatory Fortification	High Income	Americas	NA	NA
Belarus	2001	Salt	Mandatory Fortification	Upper Middle Income	Europe	NA	NA
Belgium	1992	Salt	Voluntary Fortification	High Income	Europe	NA	NA
Belize	2007	Salt	Mandatory Fortification	Upper Middle Income	Americas	85	NA

Belize	1998	Wheat Flour	Mandatory Fortification	Upper Middle Income	Americas	NA	NA
Brazil	2013	Salt	Mandatory Fortification	Upper Middle Income	Americas	98	NA
Brazil	2017	Wheat Flour	Mandatory Fortification	Upper Middle Income	Americas	NA	NA
Brazil	2002	Maize Flour	Mandatory Fortification	Upper Middle Income	Americas	NA	NA
Brunei Darussalam	2001	Salt	Voluntary Fortification	High Income	Asia	NA	NA
Bulgaria	2001	Salt	Mandatory Fortification	Upper Middle Income	Europe	92	NA
Canada	2017	Salt	Mandatory Fortification	High Income	Americas	NA	NA
Canada	2016	Wheat Flour	Mandatory Fortification	High Income	Americas	NA	NA
Chile	1996	Salt	Mandatory Fortification	High Income	Americas	NA	NA
Chile	2014	Wheat Flour	Mandatory Fortification	High Income	Americas	NA	NA
China, People's Republic of	2011	Salt	Mandatory Fortification	Upper Middle Income	Asia	96	NA
China, People's Republic of	2012	Oil	Voluntary Fortification	Upper Middle Income	Asia	NA	NA
China, People's Republic of	2012	Wheat Flour	Voluntary Fortification	Upper Middle Income	Asia	NA	89
Colombia	1996	Salt	Mandatory Fortification	Upper Middle Income	Americas	88	NA
Colombia	1996	Wheat Flour	Mandatory Fortification	Upper Middle Income	Americas	NA	NA
Costa Rica	2001	Salt	Mandatory Fortification	Upper Middle Income	Americas	NA	NA
Costa Rica	2001	Wheat Flour	Mandatory Fortification	Upper Middle Income	Americas	NA	NA
Costa Rica	2006	Rice	Mandatory Fortification	Upper Middle Income	Americas	NA	NA
Costa Rica	1999	Maize Flour	Mandatory Fortification	Upper Middle Income	Americas	NA	NA

Croatia	2011	Salt	Mandatory Fortification	Upper Middle Income	Europe	NA	NA
Cuba	2012	Wheat Flour	Mandatory Fortification	Upper Middle Income	Americas	NA	NA
Cyprus	2006	Salt	Voluntary Fortification	High Income	Asia	NA	NA
Czech Republic	2006	Salt	Voluntary Fortification	High Income	Europe	NA	NA
Denmark	2014	Salt	Mandatory Fortification	High Income	Europe	NA	NA
Dominica	1992	Wheat Flour	Mandatory Fortification	Upper Middle Income	Americas	NA	NA
Dominican Republic	2009	Wheat Flour	Mandatory Fortification	Upper Middle Income	Americas	NA	NA
Dominican Republic	2009	Maize Flour	Voluntary Fortification	Upper Middle Income	Americas	NA	NA
Ecuador	2010	Salt	Mandatory Fortification	Upper Middle Income	Americas	NA	NA
Ecuador	2011	Wheat Flour	Mandatory Fortification	Upper Middle Income	Americas	NA	NA
Estonia	2006	Salt	Voluntary Fortification	High Income	Europe	NA	NA
Fiji	2009	Salt	Mandatory Fortification	Upper Middle Income	Oceania	NA	NA
Fiji	2009	Wheat Flour	Mandatory Fortification	Upper Middle Income	Oceania	NA	NA
Finland	2015	Salt	Voluntary Fortification	High Income	Europe	NA	NA
France	2007	Salt	Voluntary Fortification	High Income	Europe	NA	NA
Gabon	2004	Salt	Mandatory Fortification	Upper Middle Income	Africa	89	NA
Germany	1993	Salt	Voluntary Fortification	High Income	Europe	NA	NA
Greece	2006	Salt	Voluntary Fortification	High Income	Europe	NA	NA
Grenada	1992	Wheat Flour	Mandatory Fortification	Upper Middle Income	Americas	NA	NA

Guyana	1992	Wheat Flour	Mandatory Fortification	Upper Middle Income	Americas	NA	NA
Hungary	2013	Salt	Mandatory Fortification	High Income	Europe	NA	NA
Iran, Islamic Republic of	2006	Wheat Flour	Mandatory Fortification	Upper Middle Income	Asia	NA	NA
Iraq	2000	Wheat Flour	Mandatory Fortification	Upper Middle Income	Asia	NA	NA
Ireland	2006	Salt	Voluntary Fortification	High Income	Europe	NA	NA
Italy	1995	Salt	Mandatory Fortification	High Income	Europe	NA	NA
Jamaica	1992	Wheat Flour	Mandatory Fortification	Upper Middle Income	Americas	NA	NA
Kazakhstan	2003	Salt	Mandatory Fortification	Upper Middle Income	Asia	94	NA
Kazakhstan	2008	Wheat Flour	Mandatory Fortification	Upper Middle Income	Asia	41	100
Kuwait	2012	Salt	Mandatory Fortification	High Income	Asia	NA	NA
Kuwait	2006	Wheat Flour	Mandatory Fortification	High Income	Asia	NA	NA
Latvia	2000	Salt	Voluntary Fortification	High Income	Europe	NA	NA
Lithuania	2015	Salt	Mandatory Fortification	High Income	Europe	NA	NA
Luxembourg	2006	Salt	Voluntary Fortification	High Income	Europe	NA	NA
Macedonia, The former Yugoslav Republic of	1999	Salt	Mandatory Fortification	Upper Middle Income	Europe	NA	NA
Malaysia	1985	Salt	Voluntary Fortification	Upper Middle Income	Asia	28	NA
Malaysia	2017	Wheat Flour	Voluntary Fortification	Upper Middle Income	Asia	NA	NA
Malta	2006	Salt	Voluntary Fortification	High Income	Europe	NA	NA
Mexico	2009	Wheat Flour	Mandatory Fortification	Upper Middle Income	Americas	NA	NA

Mexico	2009	Maize Flour	Mandatory Fortification	Upper Middle Income	Americas	NA	NA
Mexico	1993	Salt	Mandatory Fortification	Upper Middle Income	Americas	NA	90
Netherlands	2008	Salt	Voluntary Fortification	High Income	Europe	NA	NA
New Zealand	2015	Salt	Mandatory Fortification	High Income	Oceania	NA	NA
Norway	2016	Salt	Voluntary Fortification	High Income	Europe	NA	NA
Oman	2010	Oil	Mandatory Fortification	High Income	Asia	NA	NA
Oman	2010	Wheat Flour	Mandatory Fortification	High Income	Asia	NA	NA
Oman	2012	Salt	Mandatory Fortification	High Income	Asia	NA	NA
Panama	2003	Wheat Flour	Mandatory Fortification	Upper Middle Income	Americas	NA	NA
Panama	2009	Rice	Mandatory Fortification	Upper Middle Income	Americas	NA	NA
Panama	2001	Salt	Mandatory Fortification	Upper Middle Income	Americas	NA	NA
Paraguay	2002	Wheat Flour	Mandatory Fortification	Upper Middle Income	Americas	NA	NA
Paraguay	2014	Salt	Mandatory Fortification	Upper Middle Income	Americas	93	NA
Peru	2006	Wheat Flour	Mandatory Fortification	Upper Middle Income	Americas	NA	NA
Peru	2006	Salt	Mandatory Fortification	Upper Middle Income	Americas	87	NA
Poland	2010	Salt	Mandatory Fortification	High Income	Europe	NA	NA
Portugal	2006	Salt	Voluntary Fortification	High Income	Europe	NA	NA
Qatar	2006	Wheat Flour	Voluntary Fortification	High Income	Asia	NA	NA
Qatar	2012	Salt	Mandatory Fortification	High Income	Asia	NA	NA

Romania	2009	Salt	Mandatory Fortification	Upper Middle Income	Europe	NA	87
Russian Federation	2001	Salt	Voluntary Fortification	Upper Middle Income	Europe	NA	NA
Saint Kitts and Nevis	1992	Wheat Flour	Mandatory Fortification	High Income	Americas	NA	NA
Saint Lucia	1992	Wheat Flour	Mandatory Fortification	Upper Middle Income	Americas	NA	NA
Saint Vincent and the Grenadines	1992	Wheat Flour	Mandatory Fortification	Upper Middle Income	Americas	NA	NA
Saudi Arabia	2006	Wheat Flour	Mandatory Fortification	High Income	Asia	NA	NA
Saudi Arabia	2012	Salt	Mandatory Fortification	High Income	Asia	NA	NA
Singapore	2006	Salt	Voluntary Fortification	High Income	Asia	NA	NA
Slovakia	2005	Salt	Mandatory Fortification	High Income	Europe	NA	NA
Slovenia	1998	Salt	Mandatory Fortification	High Income	Europe	NA	NA
South Africa	2008	Wheat Flour	Mandatory Fortification	Upper Middle Income	Africa	NA	NA
South Africa	2008	Maize Flour	Mandatory Fortification	Upper Middle Income	Africa	NA	NA
South Africa	2006	Salt	Mandatory Fortification	Upper Middle Income	Africa	NA	NA
Spain	1983	Salt	Voluntary Fortification	High Income	Europe	NA	NA
Suriname	1992	Wheat Flour	Mandatory Fortification	Upper Middle Income	Americas	NA	NA
Sweden	1983	Salt	Voluntary Fortification	High Income	Europe	NA	NA
Switzerland	2005	Salt	Voluntary Fortification	High Income	Europe	NA	NA
Thailand	2011	Salt	Mandatory Fortification	Upper Middle Income	Asia	85	NA
Trinidad and Tobago	1992	Wheat Flour	Mandatory Fortification	High Income	Americas	NA	NA

Turkey	2013	Salt	Mandatory Fortification	Upper Middle Income	Asia	85	NA
Turkmenistan	2006	Wheat Flour	Mandatory Fortification	Upper Middle Income	Asia	NA	NA
Turkmenistan	2002	Salt	Mandatory Fortification	Upper Middle Income	Asia	100	NA
United Arab Emirates	2006	Wheat Flour	Voluntary Fortification	High Income	Asia	NA	NA
United Arab Emirates	2012	Salt	Mandatory Fortification	High Income	Asia	NA	NA
United Kingdom of Great Britain and Northern Ireland	1998	Wheat Flour	Mandatory Fortification	High Income	Europ e	NA	NA
United Kingdom of Great Britain and Northern Ireland	2006	Salt	Voluntary Fortification	High Income	Europ e	NA	NA
United States of America	2017	Wheat Flour	Mandatory Fortification	High Income	Amer icas	NA	NA
United States of America	2017	Rice	Mandatory Fortification	High Income	Amer icas	NA	NA
United States of America	2017	Maize Flour	Mandatory Fortification	High Income	Amer icas	NA	NA
United States of America	2016	Salt	Voluntary Fortification	High Income	Amer icas	NA	NA
Uruguay	2006	Wheat Flour	Mandatory Fortification	High Income	Amer icas	NA	NA
Uruguay	1990	Salt	Mandatory Fortification	High Income	Amer icas	NA	NA
Venezuela, Bolivarian Republic of	2001	Wheat Flour	Mandatory Fortification	Upper Middle Income	Amer icas	NA	NA
Venezuela, Bolivarian Republic of	1996	Maize Flour	Mandatory Fortification	Upper Middle Income	Amer icas	NA	NA
Venezuela, Bolivarian Republic of	1993	Rice	Voluntary Fortification	Upper Middle Income	Amer icas	NA	NA
Venezuela, Bolivarian Republic of	1995	Salt	Mandatory Fortification	Upper Middle Income	Amer icas	NA	NA

Source: Global Fortification Data Exchange. [Accessed 06/03/2019] http://www.fortificationdata.org. Notes: The year reflects the most updated legislation (mandatory) or voluntary fortification initiative rather than the year when this was first introduced (- used where no year was present). NA denotes no data available. Countries with an unknown status i.e. mandatory/voluntary fortification have been excluded. Coverage refers to the proportion of the fortified vehicle being consumed from the overall population i.e. percent of households found through quantitative analyses to be consuming a fortified produce. Proportion industrially processed reflects

the proportion of the food vehicle in the country that is industrially processed and thus presents an opportunity for fortification. A food vehicle that has been industrially processed is defined as "processed in facilities of the following capacity: (i) Wheat and Maize Flours – 20 MT/day grain processing rated capacity; (ii) Rice – 5 MT hour paddy processing rated capacity; (iii) Oil – 5 MT/day rated capacity. (iv) Salt – 5,000 MT/year raw salt rated capacity" (See Global fortification Data Exchange). The same notes apply to Tables A2 and A3.

Table S2. Lower middle income by regulation type, food vehicle and household coverage/proportion industrially processed.

Country	Year	Food Vehicle	Indicator	Income Status	Region	Coverage of industrially produced, fortified /total population (%)	proportion industrially processed (%)
Angola	2006	Salt	Mandatory Fortification	Lower Middle Income	Africa	82	NA
Armenia	2001	Salt	Mandatory Fortification	Lower Middle Income	Asia	99	NA
Bangladesh	1989	Salt	Mandatory Fortification	Lower Middle Income	Americas	69	89
Bangladesh	2013	Oil	Mandatory Fortification	Lower Middle Income	Americas	NA	52
Bangladesh	(Blanks)	Rice	Voluntary Fortification	Lower Middle Income	Americas	NA	NA
Bolivia, Plurinational State of	2013	Salt	Mandatory Fortification	Lower Middle Income	Americas	86	NA
Bolivia, Plurinational State of	2009	Oil	Mandatory Fortification	Lower Middle Income	Americas	NA	NA
Bolivia, Plurinational State of	2011	Wheat Flour	Mandatory Fortification	Lower Middle Income	Americas	NA	NA
Cabo Verde	2004	Salt	Mandatory Fortification	Lower Middle Income	Africa	97	NA
Cabo Verde	2014	Wheat Flour	Mandatory Fortification	Lower Middle Income	Africa	NA	100
Cambodia	2007	Salt	Mandatory Fortification	Lower Middle Income	Asia	68	69
Cameroon	1995	Salt	Mandatory Fortification	Lower Middle Income	Africa	86	NA
Cameroon	2011	Wheat Flour	Mandatory Fortification	Lower Middle Income	Africa	NA	NA
Congo	2004	Salt	Mandatory Fortification	Lower Middle Income	Africa	93	NA

Congo	2013	Wheat Flour	Mandatory Fortification	Lower Middle Income	Africa	NA	NA
Cote d'Ivoire	2007	Oil	Mandatory Fortification	Lower Middle Income	Africa	NA	NA
Cote d'Ivoire	2007	Wheat Flour	Mandatory Fortification	Lower Middle Income	Africa	NA	98
Cote d'Ivoire	2001	Salt	Mandatory Fortification	Lower Middle Income	Africa	80	NA
Djibouti	2013	Oil	Mandatory Fortification	Lower Middle Income	Africa	NA	NA
Djibouti	2013	Wheat Flour	Mandatory Fortification	Lower Middle Income	Africa	NA	100
Egypt	(Blanks)	Wheat Flour	Mandatory Fortification	Lower Middle Income	Africa	NA	100
Egypt	2015	Salt	Mandatory Fortification	Lower Middle Income	Africa	93	NA
El Salvador	2005	Salt	Mandatory Fortification	Lower Middle Income	Americas	NA	NA
El Salvador	2007	Wheat Flour	Mandatory Fortification	Lower Middle Income	Americas	NA	NA
El Salvador	2008	Maize Flour	Mandatory Fortification	Lower Middle Income	Americas	NA	NA
Georgia	2014	Salt	Mandatory Fortification	Lower Middle Income	Asia	100	NA
Ghana	2006	Salt	Mandatory Fortification	Lower Middle Income	Africa	57	18
Ghana	2006	Oil	Mandatory Fortification	Lower Middle Income	Africa	NA	NA
Ghana	2006	Wheat Flour	Mandatory Fortification	Lower Middle Income	Africa	NA	100
Guatemala	2004	Salt	Mandatory Fortification	Lower Middle Income	Americas	88	NA
Guatemala	2001	Wheat Flour	Mandatory Fortification	Lower Middle Income	Americas	NA	NA
Guatemala	2016	Maize Flour	Mandatory Fortification	Lower Middle Income	Americas	NA	NA
Honduras	1960	Salt	Mandatory Fortification	Lower Middle Income	Americas	NA	NA

Honduras	2007	Wheat Flour	Mandatory Fortification	Lower Middle Income	Americas	NA	NA
India	2016	Salt	Mandatory Fortification	Lower Middle Income	Asia	93	NA
India	2016	Oil	Voluntary Fortification	Lower Middle Income	Asia	NA	NA
India	2016	Wheat Flour	Voluntary Fortification	Lower Middle Income	Asia	NA	NA
India	2016	Rice	Voluntary Fortification	Lower Middle Income	Asia	NA	NA
Indonesia	2010	Salt	Mandatory Fortification	Lower Middle Income	Asia	92	NA
Indonesia	2009	Wheat Flour	Mandatory Fortification	Lower Middle Income	Asia	NA	NA
Indonesia	2012	Oil	Voluntary Fortification	Lower Middle Income	Asia	NA	NA
Jordan	2002	Wheat Flour	Mandatory Fortification	Lower Middle Income	Asia	NA	NA
Jordan	2012	Salt	Mandatory Fortification	Lower Middle Income	Asia	88	NA
Kenya	2013	Salt	Mandatory Fortification	Lower Middle Income	Africa	95	NA
Kenya	2011	Oil	Mandatory Fortification	Lower Middle Income	Africa	NA	NA
Kenya	2012	Wheat Flour	Mandatory Fortification	Lower Middle Income	Africa	NA	NA
Kenya	2012	Maize Flour	Mandatory Fortification	Lower Middle Income	Africa	NA	NA
Kiribati	2016	Salt	Mandatory Fortification	Lower Middle Income	Oceania	NA	NA
Kiribati	2014	Wheat Flour	Mandatory Fortification	Lower Middle Income	Oceania	NA	NA
Kosovo, Republic of	2008	Salt	Mandatory Fortification	Lower Middle Income	Europe	NA	NA
Kosovo, Republic of	2013	Wheat Flour	Mandatory Fortification	Lower Middle Income	Europe	NA	NA
Kyrgyzstan	2009	Wheat Flour	Mandatory Fortification	Lower Middle Income	Asia	NA	NA

Kyrgyzstan	2012	Salt	Mandatory Fortification	Lower Middle Income	Asia	99	NA
Lao People's Democratic Republic	2004	Salt	Mandatory Fortification	Lower Middle Income	Asia	80	NA
Lesotho	1999	Salt	Mandatory Fortification	Lower Middle Income	Africa	85	NA
Mauritania	2010	Oil	Mandatory Fortification	Lower Middle Income	Africa	NA	NA
Mauritania	2010	Wheat Flour	Mandatory Fortification	Lower Middle Income	Africa	NA	95
Mauritania	2004	Salt	Mandatory Fortification	Lower Middle Income	Africa	24	NA
Moldova, Republic of	2011	Salt	Mandatory Fortification	Lower Middle Income	Europ e	58	NA
Moldova, Republic of	2012	Wheat Flour	Mandatory Fortification	Lower Middle Income	Europ e	NA	NA
Mongolia	2001	Salt	Mandatory Fortification	Lower Middle Income	Asia	80	NA
Morocco	2006	Wheat Flour	Mandatory Fortification	Lower Middle Income	Africa	NA	NA
Morocco	2009	Salt	Mandatory Fortification	Lower Middle Income	Africa	43	NA
Myanmar	2011	Salt	Mandatory Fortification	Lower Middle Income	Asia	81	NA
Nicaragua	2007	Wheat Flour	Mandatory Fortification	Lower Middle Income	Ameri cas	NA	NA
Nicaragua	2014	Rice	Mandatory Fortification	Lower Middle Income	Ameri cas	NA	NA
Nicaragua	2010	Salt	Mandatory Fortification	Lower Middle Income	Ameri cas	NA	NA
Nigeria	2014	Wheat Flour	Mandatory Fortification	Lower Middle Income	Africa	NA	100
Nigeria	2014	Maize Flour	Mandatory Fortification	Lower Middle Income	Africa	NA	NA
Nigeria	2000	Oil	Voluntary Fortification	Lower Middle Income	Africa	NA	NA
Nigeria	2005	Salt	Mandatory Fortification	Lower Middle Income	Africa	93	20

Pakistan	2003	Oil	Mandatory Fortification	Lower Middle Income	Asia	NA	NA
Pakistan	2003	Oil	Voluntary Fortification	Lower Middle Income	Asia	NA	NA
Pakistan	2008	Salt	Voluntary Fortification	Lower Middle Income	Asia	69	NA
Palestine, State of	2010	Wheat Flour	Mandatory Fortification	Lower Middle Income	Asia	88	NA
Papua New Guinea	2007	Rice	Mandatory Fortification	Lower Middle Income	Ocean ia	NA	NA
Papua New Guinea	2007	Oil	Voluntary Fortification	Lower Middle Income	Ocean ia	NA	NA
Papua New Guinea	2007	Salt	Mandatory Fortification	Lower Middle Income	Ocean ia	NA	NA
Philippines	2000	Oil	Mandatory Fortification	Lower Middle Income	Asia	NA	NA
Philippines	2000	Wheat Flour	Mandatory Fortification	Lower Middle Income	Asia	NA	NA
Philippines	2000	Rice	Mandatory Fortification	Lower Middle Income	Asia	NA	NA
Philippines	2013	Salt	Mandatory Fortification	Lower Middle Income	Asia	52	NA
Sao Tome and Principe	1996	Salt	Mandatory Fortification	Lower Middle Income	Africa	91	NA
Solomon Islands	2010	Wheat Flour	Mandatory Fortification	Lower Middle Income	Ocean ia	NA	NA
Solomon Islands	2010	Salt	Mandatory Fortification	Lower Middle Income	Ocean ia	98	NA
Sri Lanka	2005	Salt	Mandatory Fortification	Lower Middle Income	Asia	95	NA
Tajikistan	2001	Salt	Mandatory Fortification	Lower Middle Income	Asia	84	NA
Tunisia	1995	Salt	Mandatory Fortification	Lower Middle Income	Africa	NA	NA
Ukraine	2015	Salt	Voluntary Fortification	Lower Middle Income	Europ e	36	NA
Uzbekistan	2005	Wheat Flour	Mandatory Fortification	Lower Middle Income	Asia	NA	NA

Uzbekistan	2001	Salt	Mandatory Fortification	Lower Middle Income	Asia	82	NA
Viet Nam	2011	Oil	Mandatory Fortification	Lower Middle Income	Asia	NA	NA
Viet Nam	2016	Wheat Flour	Mandatory Fortification	Lower Middle Income	Asia	NA	NA
Viet Nam	2011	Salt	Mandatory Fortification	Lower Middle Income	Asia	61	NA
Yemen	2001	Oil	Mandatory Fortification	Lower Middle Income	Asia	NA	NA
Yemen	2001	Wheat Flour	Mandatory Fortification	Lower Middle Income	Asia	NA	NA
Yemen	2003	Salt	Mandatory Fortification	Lower Middle Income	Asia	49	NA
Zambia	2001	Salt	Mandatory Fortification	Lower Middle Income	Africa	88	NA

Source: Global Fortification Data Exchange. [Accessed 06/03/2019] http://www.fortificationdata.org.

Table S3. Low income countries by regulation type, food vehicle and household coverage/proportion industrially processed.

Country	Year	Food Vehicle	Indicator	Income Status	Region	Coverage of industrially produced, fortified /total population (%)	Proportion industrially processed (%)
Afghanistan	2014	Salt	Mandatory Fortification	Low Income	Asia	57	NA
Afghanistan	2014	Oil	Voluntary Fortification	Low Income	Asia	33	100
Afghanistan	2014	Wheat Flour	Voluntary Fortification	Low Income	Asia	NA	92
Benin	2013	Salt	Mandatory Fortification	Low Income	Africa	69	NA
Benin	2010	Wheat Flour	Mandatory Fortification	Low Income	Africa	NA	100
Burkina Faso	2003	Salt	Mandatory Fortification	Low Income	Africa	92	NA
Burkina Faso	2010	Oil	Mandatory Fortification	Low Income	Africa	NA	NA
Burkina Faso	2010	Wheat Flour	Mandatory Fortification	Low Income	Africa	NA	NA

Burundi	2013	Salt	Mandatory Fortification	Low Income	Africa	89	NA
Burundi	2011	Oil	Mandatory Fortification	Low Income	Africa	NA	NA
Burundi	2015	Wheat Flour	Mandatory Fortification	Low Income	Africa	NA	NA
Burundi	2015	Maize Flour	Mandatory Fortification	Low Income	Africa	NA	NA
Central African Republic	1995	Salt	Mandatory Fortification	Low Income	Africa	84	NA
Chad	1994	Salt	Mandatory Fortification	Low Income	Africa	77	NA
Democratic Republic of the Congo	2003	Salt	Voluntary Fortification	Low Income	Africa	82	NA
Ethiopia	2016	Salt	Mandatory Fortification	Low Income	Africa	86	NA
Ethiopia	2018	Oil	Voluntary Fortification	Low Income	Africa	NA	NA
Ethiopia	2017	Wheat Flour	Voluntary Fortification	Low Income	Africa	NA	NA
Gambia, Republic of The	2006	Salt	Mandatory Fortification	Low Income	Africa	69	NA
Guinea	2013	Salt	Mandatory Fortification	Low Income	Africa	73	NA
Guinea	2006	Wheat Flour	Mandatory Fortification	Low Income	Africa	NA	100
Guinea-Bissau	2004	Salt	Mandatory Fortification	Low Income	Africa	26	NA
Haiti	2017	Oil	Mandatory Fortification	Low Income	Americas	NA	NA
Haiti	1992	Wheat Flour	Mandatory Fortification	Low Income	Americas	NA	NA
Haiti	2017	Salt	Mandatory Fortification	Low Income	Americas	16	NA
Liberia	2014	Salt	Mandatory Fortification	Low Income	Africa	91	NA
Liberia	2014	Oil	Mandatory Fortification	Low Income	Africa	NA	NA

Liberia	2016	Wheat Flour	Mandatory Fortification	Low Income	Africa	NA	100
Madagascar	2014	Salt	Mandatory Fortification	Low Income	Africa	68	NA
Malawi	2011	Oil	Mandatory Fortification	Low Income	Africa	NA	NA
Malawi	2011	Wheat Flour	Mandatory Fortification	Low Income	Africa	NA	NA
Malawi	2011	Maize Flour	Mandatory Fortification	Low Income	Africa	NA	NA
Malawi	1998	Salt	Mandatory Fortification	Low Income	Africa	78	NA
Mali	2010	Wheat Flour	Mandatory Fortification	Low Income	Africa	NA	100
Mali	1999	Salt	Mandatory Fortification	Low Income	Africa	90	NA
Mozambique	2012	Oil	Mandatory Fortification	Low Income	Africa	NA	NA
Mozambique	2012	Wheat Flour	Mandatory Fortification	Low Income	Africa	NA	100
Mozambique	2012	Maize Flour	Mandatory Fortification	Low Income	Africa	NA	30
Mozambique	2016	Salt	Mandatory Fortification	Low Income	Africa	42	NA
Nepal	2011	Wheat Flour	Mandatory Fortification	Low Income	Asia	37	43
Nepal	2001	Salt	Mandatory Fortification	Low Income	Asia	94	NA
Niger	2010	Wheat Flour	Mandatory Fortification	Low Income	Africa	NA	NA
Niger	2014	Salt	Mandatory Fortification	Low Income	Africa	59	NA
Rwanda	2011	Oil	Voluntary Fortification	Low Income	Africa	NA	NA
Rwanda	2010	Maize Flour	Voluntary Fortification	Low Income	Africa	NA	NA
Senegal	2013	Oil	Mandatory Fortification	Low Income	Africa	34	NA

Senegal	20<br>13	Wheat<br>Flour	Mandatory<br>Fortification	Low<br>Income	Africa	51	100
Senegal	20<br>00	Salt	Mandatory<br>Fortification	Low<br>Income	Africa	57	NA
Sierra Leone	20<br>10	Salt	Voluntary<br>Fortification	Low<br>Income	Africa	74	NA
Sierra Leone	20<br>10	Oil	Voluntary<br>Fortification	Low<br>Income	Africa	NA	NA
Sierra Leone	20<br>10	Wheat<br>Flour	Voluntary<br>Fortification	Low<br>Income	Africa	NA	100
Tanzania, United<br>Republic of	20<br>11	Oil	Mandatory<br>Fortification	Low<br>Income	Africa	54	NA
Tanzania, United<br>Republic of	20<br>10	Wheat<br>Flour	Mandatory<br>Fortification	Low<br>Income	Africa	33	98
Tanzania, United<br>Republic of	20<br>11	Maize<br>Flour	Mandatory<br>Fortification	Low<br>Income	Africa	2.5	NA
Tanzania, United<br>Republic of	20<br>13	Salt	Mandatory<br>Fortification	Low<br>Income	Africa	76	NA
Togo	20<br>13	Oil	Mandatory<br>Fortification	Low<br>Income	Africa	NA	NA
Togo	20<br>13	Wheat<br>Flour	Mandatory<br>Fortification	Low<br>Income	Africa	NA	NA
Togo	20<br>17	Salt	Mandatory<br>Fortification	Low<br>Income	Africa	77	NA
Uganda	20<br>11	Oil	Mandatory<br>Fortification	Low<br>Income	Africa	54	90
Uganda	20<br>16	Wheat<br>Flour	Mandatory<br>Fortification	Low<br>Income	Africa	8.5	100
Uganda	20<br>06	Maize<br>Flour	Mandatory<br>Fortification	Low<br>Income	Africa	6.5	30
Uganda	20<br>13	Salt	Mandatory<br>Fortification	Low<br>Income	Africa	91	NA
Zimbabwe	20<br>16	Oil	Mandatory<br>Fortification	Low<br>Income	Africa	NA	50
Zimbabwe	20<br>16	Wheat<br>Flour	Mandatory<br>Fortification	Low<br>Income	Africa	NA	40
Zimbabwe	20<br>16	Maize<br>Flour	Mandatory<br>Fortification	Low<br>Income	Africa	NA	40

Zimbabwe	2016	Salt	Mandatory Fortification	Low Income	Africa	93	NA

Source Global Fortification Data Exchange. [Accessed 06/03/2019] http://www.fortificationdata.org.

Table S4. Review of key knowledge gaps/policies/ business models by vehicle/region and scale.

References	Scale	mandatory	Voluntary	Knowledge gaps and key policies/business models identified	Vehicle											Region/country					
					1	2	3	4	5	6	7	8	9	10	11	A	B	C	D	E	F
[72]	Large scale mandatory	x	x	None of the programs assessed had predefined criteria for program coverage or coverage related to vulnerable groups Specific business model not assessed.	x	x	x											Ivory Coast, Uganda, Tanzania, South Africa, Nigeria, Senegal	Bangladesh, India		
[72]	Large scale mandatory	x		Criteria met for only 2 programmes in South Africa i.e. raw coverage, met need (reaching vulnerable population) and overall coverage ratio.		x	x											South Africa and Ivory Coast			
[72]	Large scale mandatory	x		Effective monitoring and evaluation (ongoing), particularly regulatory monitoring of the fortification process, is needed as failure to fortify i.e. vehicle not fortifiable along with choice of food vehicle were the main program bottlenecks.	x	x	x											Ivory Coast, Uganda, Tanzania, South Africa, Nigeria, Senegal	Bangladesh, India		
[35]	Large scale mandatory	x		Effective LSFF requires detailed diagnostic work including the appropriate selection of vehicles with the potential for high coverage in the population and adequate use of the fortificant. The capital-intensive startup phase of these programs means high risk. Funding not sustainable for large scale fortification programmes (e.g. 80% of government officials reported and this impacts quality control and compliance)										X							

[35]	Large-scale mandatory	X	Food lawas and regulations often fragmented and not combined within ‘legal frameworks’. Weak enforcement, monitoring of nutritional status and reach target groups currently a problem in all countries. Another criticism of mass fortification programs is one of accessibility i.e. fortified products may not reach those most in need (poorest segments of society). This may be in part because in some countries import duties/taxes on premixes or fortification equipment can result in higher prices for fortified produce. One avenue suggested to reach the poorest could be including fortified produce in social protection programs.		X			
[94]	Large-scale mandatory	X	Four key lessons for successful programs were learned: (1) choice of food fortification vehicle is imperative (2) data driven i.e. choice of food fortification dependent on the magnitude of inadequate intake/deficiency. In addition, coordination with other programs necessary i.e. food fortification programs should be part of national micronutrient deficiency control strategies; (3) fortification levels (quality compliance) needed which requires strengthening capacity/resources, governance mechanisms and policy commitment; and (4), periodic reviews of dietary patterns needed to ensure safe and impactful programs.		X			
[69]	Large-scale mandatory	x	Fortification legislation/standards set. All projects were supported with fortification equipment e.g. fortification feeders. Other mechanisms included Uzbekistan subsidizing premix as a key incentive. readjustment of plans i.e. ongoing monitoring also a key lesson. Quality of fortified foods was a common challenge experienced across the projects. Key success cited wheat flour in Morocco and Uzbekistan.	X	X	Moroc co	Vietnam	Uzbekis tan

[12]	Small scale			A study cited in Hoogendoor et al., 2017 carried out by UNCF/Gain and other partners review country experiences in small-scale salt fortification, (and wheat and maize fortification to a lesser degree). Gaps and challenges for this type o food fortification,include the proposition of **‘industry consolidation or quasi-consolidation in the form of cooperatives’**. Moreover, the need for appropriate models/business plan development and an understanding social impact also cited as a challenge.	X	x		x				
[68]	Large scale mandatory			Fragmented rice industry (11,000 millers) in Philippines compared to 11 millers i.e. universal coverage of rice fortification in Costa Rica (strong enforcement of regulation) but Philippines only successful at provincial level (5% coverage) not able to enforce legislation. Philippines had to import fortified rice due to only a small proportion of the domestic supply being fortified. Challenges consumers social marketing due to consumers feeling the fortified rice is inferior or for the poor.					x	Co sta Ric a		Philippi nes
[70]	Large scale mandatory	X		Inadequate fortification premix at industry level was found due to technological/other constraints or the quality of the premixes. Ineffective monitoring of compliance by the regulators, and self-regulation.	X	x	x	x			Nigeri a	
[73]	medium/large scale voluntary (state level)		X	Donor/govt provision of technical and financial assistance at different stages and helped to create a partnership between the private sector (Gujarat Roller Flour Millers Association (GRFMA)) The government then agreed to swap wheat grain for what flour in its Social Safety Net Programmes (SSNPs). Pilot testing and prior examination of risk a priority.	X							

[77]	Large scale voluntary	x		Selection of vehicle and micronutrients needed were done prior as well as engagement with industry to assess willingness to participate. Chosen production units were equipped with necessary equipment for fortification. Training of staff on fortification and quality contrl was done as was social marketing and communication activities were carried out as per the strategy developed. Other successes included the formation of a state food fortification alliance was formed in Madhya Pradesh with all relevant stakeholders. High compliance rates were found.	X		x		x			India	
[37]	Small-scale			Machinery for small-scale iodisation of salt was found not to be in use. Problems of high running costs and training. Improved low cost method of knack spraying with close supervision resulted in improved levels in iodine levels in salt thus a promising method in low resource settings. Continuous training, monitoring and enforcement, is also needed.				x			Tanza nia		
.[36]	Small-scale	x		Where market segmentation of products is well established (i.e. hammer, breakfast, roller maize meals) there may be scope of introducing fortification through improving the capacity of a subset of hammer mills in the country (commonly used by households); though given the geographic locations and costs involved in training and quality assurance this is unlikely to be an option on a large-scale.		x					Zambi a		
[26]	Large-scale Voluntary		x	Likewise, in the Philippines, voluntary fortification for staples (e.g. Wheat flour and oil) led to products being out of reach for ordinary Filipino families because of the higher cost .	X		x						Philippi nes
[12]	Large scale voluntary		x	Likewise, successes with voluntary fortification have been found in South East Asia (e.g. Philippines and Cambodia) particularly with fortification of sauces, processed foods and seasonings due to centralised production and there being only two manufacturers in the country with one controlling 90% of						x			Philippi nes and Cambod ia

				the market share (Hoogendoorn et al., 2017).				
[26]	Large scale	x	x	The successful case of the 77 countries that mandated wheat flour fortification, for example, showed that 90% of these had industrially milled flour compared to only 5% of flour (industrially milled) where countries had gone through fortification voluntarily .	X			
[32]	Large scale mandatory	x		Major challenges was issue with the premix at mill level e.g. starch filler, tended to harden and break the helix (used to stir the premix to avoid lumping) on the microfeeder (alternative found). 11 oil factories involved in fortification of oil the tender process for the supply of premix and the fortification units is under way and is fully funded by the Government of Egypt. Financial sustainability is likely to be an issue.	X	x		Egypt
[34]	Large scale mandatory	x		Greiner [34] also suggested that staple foods in particular should be fortified through mandatory legislation. The need for developing capacities in assessment and monitoring to ensure the right concentrations of nutrients are added are seen as a priority			x	
[27]	Large scale mandatory	x		Whilst largely successful in many industrialised countries two major challenges remain: (i) the ability to guarantee that standards of nutrient quality are met, and, (ii) universal coverage through mass fortification not to exclude the most vulnerable members of society.				

[30]	Large scale		x	Where voluntarily fortification has taken place this has often resulted in a failure to reach scale/target groups. High capital investment costs have deterred millers from switching to expensive coating technology. Spraying methods used have only resulted in moderate coverage. Millers are unwilling to switch to more proven methods, such as extrusion or coating, which will incur higher capital investment and recurring costs. Policy makers need to improve standards and regulatory capacity in order for rice fortification to be effective.	X			Colum bia		
[67]	Large scale mandatory	x		Public sector-led fortification programs, have been successful in a number of countries largely as a result of mandatory fortification (legislation), enforceable regulation and a mixture of incentives. Voluntary fortification efforts still challenging for staple foods given the lack of ability to bring to scale. social marketing be founded on a strong category brand identity and engagement from the public and social sectors needed. Budget, time, and reach constraints also constraints.	x			Brazil		
[71]	Large scale mandatory	x		Higher price of fortified produce may deter consumers and lack of engagement of private sector flour mills given the purchase of maize from informal markets.					Tanza nia	
[75]	Voluntary fortification		x	affordability being the main reason that consumers did not purchase the product (yogurt), however, challenges in marketing (particularly in facilitating repeat purchasers) and distribution were also cited as significant challenges.		x				Bangladesh
[76]	Voluntary fortification		x	Market driven approaches such as the case of Tiger biscuits (aimed at poorer households) in India have also been unsuccessful in reaching scale; due in part to low awareness among poor consumers, cheap alternative foods and a unfavourable institutional environment e.g. lack of food safety standards/regulation			x			

[35]	Large scale mandatory	x	Guatemala, Honduras, and El Salvador early in the 1970s. Sugar chose because it was a food vehicle consumed by population at large including vulnerable grous and its industrialized, centralized processing that facilitated. Close supervision and. Public–private partnerships were established to work toward the establishment of national programs. This wa supported by careful advocacy/promotional programs and mandatory legislation.						Guatemala, Honduras, and El Salvador		
[81]	Small-medium scale		regular training needed as limited skills of community health workers and.maintaining the supply chain of micronutrient powders and demonstrating impact are key to being able to bring to scale					x			Bangladesh
[79]	Small-medium scale		Different packaging sizes and price points, which helped to adapt to specific disposable income as well as local manufacture of the micronutrient powder, was a key feature of the success of this strategy. (1) scale across all provinces in the country requires forecasting/planning for distribution; (2) behaviour change activities in tandem (3) design a complementary delivery model in order to reach to vulnerable groups, e.g. ethnic minorities/geographically marginalized, Based on a thorough business plan is also needed as was done in this case.					x			
[78]	Small-scale	x	Feasible/sustainable options for premix supply needed, as is quality control, and the need to ensure cost recovery. Small-scale demanding at the community level and thus encourage medium scale initiative to support home fortification may be an option as is bringing such efforts into nation fortification frameworks (especially in countries with populations with very low access to fortified foods).	x	x	x				Malawi, Tanzania and Senegal	
[95]	Large scale mandatory	x	Need to harmonise cross border standards especially neighbouring countries with similar conditions which would reduce conflict between health/trade. Choice of food vehicle important (i.e. consumed by majority of				X				

			population) and centralized production with few produce. The fortified produce must also not change i.e. sensorially so as to deter consumers. Effective monitoring and evaluation data on coverage quality, coverage needed as failure often relate to lack of industry buy-in and government political will and regulatory enforcement capacity, which is reflected in inefficient or non-existent quality control.							
Garrett et al., (2018)			National alliances needed as the play a critical role of oversight and guidance in order to improve fortification programs					x		
[85]			Four levels are reported ranging from an individual company to a multi-stakeholder institution. Level 1 relates to cooperation with business partners along value chains (this involves minimum involvement from the private sector to sustainable development including nutrition). Level 2 is situated at the project-level i.e. financing and implementation of partnerships; small scale companies linking with other bodies such as investors, governments, philanthropic organisations, NGOs and research centres. Level 3 is industry-level precompetitive business alliances: association of companies: industries and corporate bodies (this is a more organised approach towards sustainable development). Finally, level 4 consists of multi-stakeholder institution platforms and networks: those can be formal or informal platforms					X		
[39]			Ultra Rice has shown success in reducing iron deficiency but needs to be tested on a larger scale for inclusion into large scale micronutrient programs.				X		Mexico	
[87]	Large scale/small scale salt	x	Legal framework from Indonesia and vietnam The legal framework is a crucial factor for the success of food fortification programs, as it shapes to a large extent the implementation of food fortification. The legal framework is instrumental to ensure the quality,	x	x	x				Indonesia and Vietnam

			safety, availability, cost-effectiveness, and sustainability of food fortification. In the first place, the legal framework should specify the fortificants and fortification levels, as well as the food vehicles and the fortification procedures. In addition, it should ensure the commitment of policy makers and producers to fortification
[25]	Large scale	x	Strong political commitment including enforcement of regulation. Engaging of private sector but with strong public backing and partnerships i.e. donors medical organisations etc. social marketing to raise awareness among population and diagnostic work on the scientific merits of the intervention includes data on deficiency in relevant groups etc. awareness and promote demand. Market studies to ensure implementation goals are being delivered and/or the need to make adjustments where needed.

Notes: 1=wheat flour, 2=maize flour, 3= oil, 4=salt= 5=rice= 6=sugar….7=milk 8=yogurt 9=biscuits 10= not specified 11=seasonings/sauces; A- Central US, B=South America, C=Africa, D=Southern Asia, E=East Asia, F=Central Asia where blank the country or legislation status was not specified.

Table S5. Biofortification review of key knowledge gaps/policies/ business models.

Reference	Scale	Gaps/lessons/business model	Maize	Orange sweet potato	Wheat	Beans	Non-specified	Africa	Asia	Asia
[13, 49]	Large scale	Cumulatively, more than 150 biofortified varieties of 10 crops have been released in 30 countries to date bred through conventional plant breeding rather than transgenic (genetically modified). Scaling will building and expanding existing partnerships across the value chain. Delivery strategies have varied dependent on crop and location. Some where private seed companies have played a stronger role and public sector leading (e.g. where public sector multiplies and distributes seed). Integration of biofortification into standards e.g. Codex will also help cross border marketing distribution.	x	x	x	x		x	x	x
[83]	Large scale/small-scale	Challenge of continued supply of biofortified crops and adequate extension services. social networks increase,<br>for biofortified crops, including proximity to local seed multipliers which are likely to improve the opportunity of learning and information regarding planting materials.				x		Rwanda		
Meenakhi[10, 11]		Cost effectiveness of biofortification very high					x			
[12]		Better diagnostic work is needed in order to identify wherefortification/biofortification is likely to have an impact (e.g. where the product has already very high coverage rather than divesting resources where impact is unlikely					x			
